# Ammonia-fed reversible protonic ceramic fuel cells with Ru-based catalyst

**DOI:** 10.1038/s42004-021-00559-2

**Published:** 2021-08-17

**Authors:** Liangzhu Zhu, Chris Cadigan, Chuancheng Duan, Jake Huang, Liuzhen Bian, Long Le, Carolina H. Hernandez, Victoria Avance, Ryan O’Hayre, Neal P. Sullivan

**Affiliations:** 1 Metallurgical and Materials Engineering Department, Colorado School of Mines, Golden, CO USA; 2 Ningbo Institute of Materials Technology and Engineering, Chinese Academy of Sciences, Ningbo, China; 3 Mechanical Engineering Department, Colorado School of Mines, Golden, CO USA; 4 Chemical Engineering Department, Kansas State University, Manhattan, KS USA

**Keywords:** Fuel cells, Fuel cells

## Abstract

The intermediate operating temperatures (~400–600 °C) of reversible protonic ceramic fuel cells (RePCFC) permit the potential use of ammonia as a carbon-neutral high energy density fuel and energy storage medium. Here we show fabrication of anode-supported RePCFC with an ultra-dense (~100%) and thin (4 μm) protonic ceramic electrolyte layer. When coupled to a novel Ru-(BaO)_2_(CaO)(Al_2_O_3_) (Ru-B2CA) reversible ammonia catalyst, maximum fuel-cell power generation reaches 877 mW cm^−2^ at 650 °C under ammonia fuel. We report relatively stable operation at 600 °C for up to 1250 h under ammonia fuel. In fuel production mode, ammonia rates exceed 1.2 × 10^−8^ NH_3_ mol cm^−2^ s^−1^at ambient pressure with H_2_ from electrolysis only, and 2.1 × 10^−6^ mol NH_3_ cm^−2^ s^−1^ at 12.5 bar with H_2_ from both electrolysis and simulated recycling gas.

## Abstract (web summary)

Ammonia-fed protonic ceramic fuel cells could be a more sustainable alternative than their hydrogen-fed counterparts. In this work, the authors couple an reversible ammonia catalyst with a protonic ceramic electrochemical cell, enabling cracking of ammonia into H_2_ and N_2_ for fuel-cell mode operation, as well as synthesis of ammonia from H_2_O and N_2_ in electrolysis mode operation.

## Introduction

Protonic ceramic fuel cells (PCFCs) are an increasingly important sub-class of conventional solid oxide fuel cells (SOFCs) that primarily conduct protons, and therefore operate at lower temperatures (typically between ~400 and 650 °C) compared to their oxygen-ion conducting counterparts. PCFCs have attracted increasing attention in the past decade due to large improvements in performance^1–8^. Recent studies, along with a foundation from earlier pioneering work^9–14^, have provided major advancements, including new electrolyte compositions with working temperatures ~200 °C lower than that in typical SOFCs^5–7^, new triple-conducting cathode materials^6,8,14,15^, improvements in cell fabrication process^6,16^, and methods to manipulate interfacial properties^4^ to dramatically improve performance and stability. The recent demonstration of a 5 × 5 cm^2^ PCFC reaching 1.3 W cm^−2^ maximum power density (MPD) at 600 °C under H_2_ fuel, and the demonstration of 10 × 10 cm^2^ PCFC single-cell generating 42 W output at 600 °C suggest PCFCs may now be poised for practical application^5,17^. Moreover, when operated in reverse, most PCFCs can also be used as protonic ceramic electrolysis cells (PCECs) to produce H_2_. Recent studies have demonstrated reversible operation in PCFC/PCEC devices with close to 100% H_2_ faradaic efficiency (FE) at current densities as high as 2000 mA cm^−2^ and temperatures between 500 and 600 °C^2,18,19^.

Hydrogen is the most-common working fluid in reversible devices towards a sustainable green energy-oriented future, However, NH_3_ presents an attractive alternative. Ammonia can be easily liquefied at room temperature at about 8 bar or at −33 °C at ambient pressure. In contrast, the liquefaction temperature of hydrogen is −253 °C at ambient pressure and liquefaction of hydrogen gas at room temperature is not practical. When liquified, NH_3_ has a significantly higher volumetric energy density (12.7 MJ/L) than compressed hydrogen (4.5 MJ/L at ~70 MPa) or liquified hydrogen (8.5 MJ/L) and as well as Li-ion batteries (~5.4 MJ/L)^20^. Ammonia is a widely used raw material for agriculture fertilizer and thus has well-established storage, transport, and handling processes (about 180 million tons of ammonia are produced annually)^21,22^. Techno-economic analysis suggests ammonia is the least expensive fuel among hydrogen, gasoline, natural gas, liquified petroleum gas, and methanol^23,24^. Finally, NH_3_ has a much narrower explosive limit in air (15–28 vol. %) in contrast to compressed hydrogen (4–75 vol. %) and methanol (~7–36 vol. %), and a high auto-ignition temperature (650 °C) that reduces flammability risk during storage and transportation^24^. With these advantages in mind, an energy system that can combine the advantages of both RePCFCs and NH_3_ fuel may be very attractive for space-tight and high energy-density applications such as in fuel cell vehicles, unmanned aerial vehicles, and seasonal-term large scale energy storage.

In this study, we report a high-performance reversible PCFC coupled to a novel, highly effective and reversible Ru-(BaO)_2_(CaO)(Al_2_O_3_) (Ru-B2CA) ammonia catalyst. By integrating the Ru-B2CA catalyst with our high-performance PCFC, we achieved a peak power density of 877 mW cm^−2^ at 650 °C under NH_3_ fuel, which is among the highest performing ammonia-fed SOFCs and PCFCs at this temperature (*c.f*. Supplementary Table S1). Performance on NH_3_ reaches 93% to 98% of that achieved under H_2_ fuel from 450 to 650 °C, suggesting very little penalty for operating on NH_3_ relative to neat H_2_. Furthermore, degradation under NH_3_ fuel, a significant challenge for most fuel cells, is significantly suppressed with the aid of the Ru-B2CA catalyst. When operation is reversed to fuel-synthesis mode, the same device demonstrates exceptional performance for steam electrolysis, with high FE, current densities exceeding 2000 mAcm^−2^, and high ammonia production rates at ambient pressure as compared in Supplementary Table S2. Finally, we further demonstrate reversible cycling between ammonia production and electric-power generation in a single cell with high performance and reasonable stability.

## Results and discussions

### A decoupled ammonia power generation and ammonia synthesis design

**Fig. 1:**
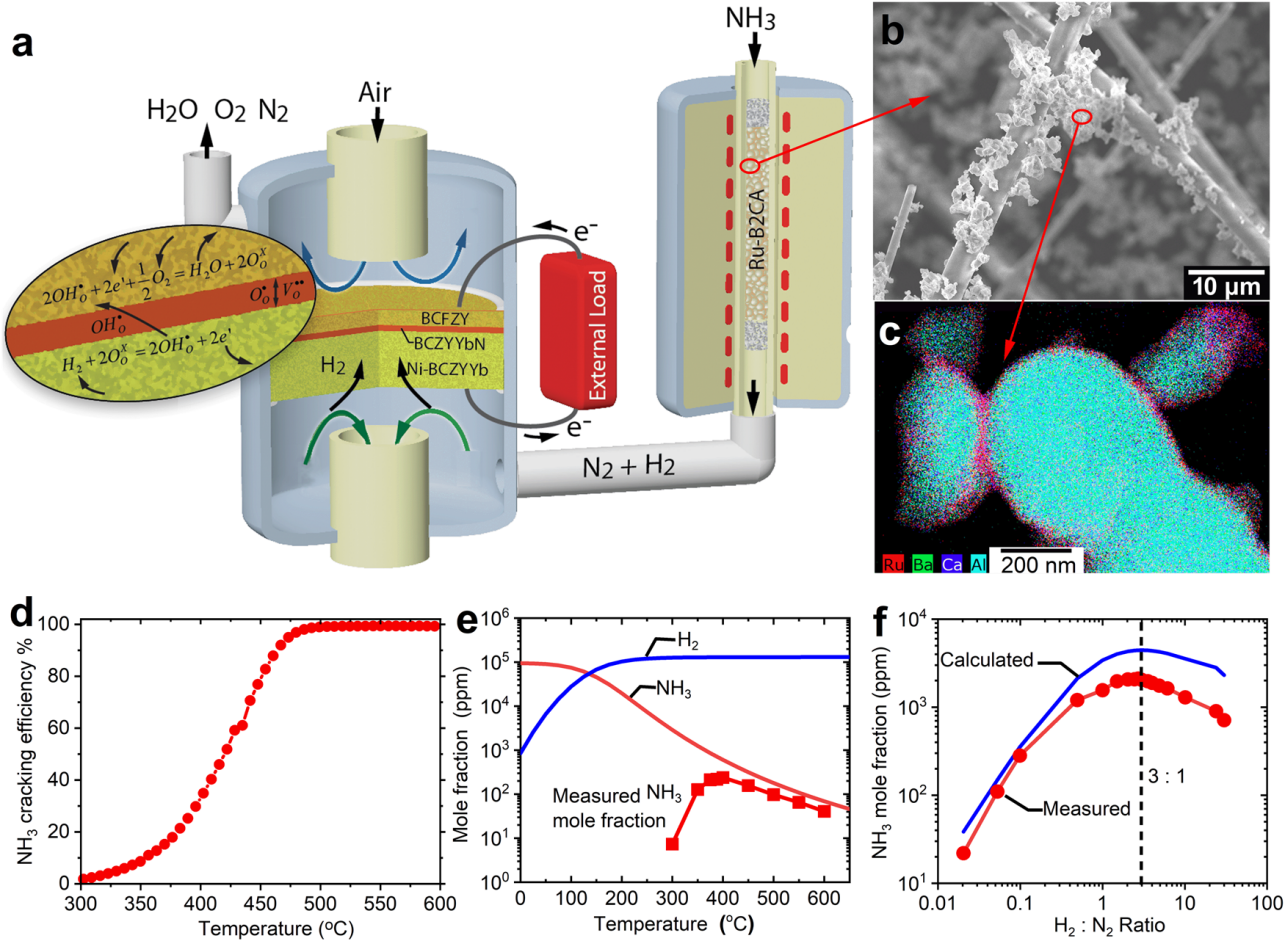
Design of decoupled ammonia power generation and production approach and characterization of the Ru-B2CA catalyst and support. **a** Schematic process flow for ammonia power generation. The process is reversible for ammonia production. **b** Ru-B2CA is mixed with refractory insulation fibers in order to facilitate mass transport and minimize pressure change in the ammonia synthesis/cracking reactor when coupling to a ceramic cell. **c** TEM-EDX elemental maps showing reasonable uniformity across 300-nm particles and evidence of Ru-B2CA core-shell structure. **d** Measured cracking efficiency of Ru-B2CA as a function of temperature. Testing conditions: 0.2 g of 1 wt. % Ru-B2CA mixed with 0.4 g of Al_2_O_3_, 10 sccm NH_3_, 1 atm pressure. **e** Measured ammonia production rate as a function of temperature with comparison to thermodynamic calculations. Testing conditions: 8 g of 2.5 wt. % Ru-B2CA mixed with 8 g of thermal insulation fibers, 100 sccm N_2_ and 15 sccm H_2_, 1 atm pressure. **f** Measured ammonia production rate as a function of H_2_ to N_2_ ratio with comparison to thermodynamic calculations. Testing conditions: 2.5 g of 5 wt. % Ru-B2CA mixed with 2.5 g of thermal insulation fibers, 100 sccm total flow rate, 1 atm pressure, reactor temperature at 400 °C.

Figure 1a illustrates our hybrid approach for ammonia power generation and ammonia production. In “energy-generation” mode, NH_3_ fuel is fed and split over the catalyst to form H_2_ and N_2_; the produced H_2_ is electrochemically oxidized to generate electricity. This approach enables full reversibility. In “energy-storage” mode, feedstocks of H_2_O, N_2_ and electricity form NH_3_ in addition to excess H_2_ and N_2_. While prior ammonia-based applications of protonic ceramic cells have largely pursued a fully-integrated, single reactor approach, here we deliberately pursue a decoupled design, where separate reactors are used for power generation/electrochemical water splitting and ammonia cracking/thermochemical ammonia synthesis.

We selected this decoupled design for several reasons. (1) Decoupling enables the size and operating conditions for each portion of the device to be independently optimized. We operate the protonic-ceramic electrolyzer at ~600 °C for efficient H_2_ production, while the NH_3_ synthesis step is conducted at ~400–500 °C to maximize ammonia production rates based on the thermodynamic and kinetic characteristics of the ammonia synthesis reaction. (2) Decoupling greatly simplifies device fabrication procedures, as integrating the non-conductive Ru-B2CA catalyst directly within the RePCEC electrode necessitates costly and performance-compromising synthesis steps. (3) The decoupling also allows the geometry and characteristics of the Ru-B2CA catalyst bed (e.g., residence time, catalyst loading, operating pressure) to be independently controlled relative to the geometry and characteristics of the electrolysis cell (e.g., H_2_ production rate, Faradaic efficiency). (4) Decoupling also facilitates periodic regeneration/recycling of the ammonia catalyst independent of the electrochemical cell. Furthermore, the decoupled design facilitates direct adoption into the high pressure and temperature synthesis loop found in the conventional HB process. (5) PCEC production of pure, dry hydrogen removes the need for the gas separation, purification and dehumidification processes required for SMR-based H_2_ production, simplifying system design. (6) Potential thermal advantages arise as the endotherm associated with steam electrolysis can be balanced by the exotherm present in ammonia synthesis; similarly, under fuel-cell mode, the NH_3-_cracking endotherm can be offset by heat produced by fuel-cell inefficiencies. (7) Finally, thermodynamic, kinetic, and catalytic analyses (further detailed in the SI) suggest it is fundamentally very challenging to suppress hydrogen-evolution reaction while increasing the nitrogen reduction reaction^25–28^. Using a decoupled design, ammonia catalysis and H_2_ evolution can be spatially separated and therefore independently optimized without compromise.

We note that our decoupled design approach is in contrast to most previous attempts to use protonic ceramic electrochemical devices for ammonia synthesis, as in the pioneering work of the Stoukides group^29,30^, where direct integration of the ammonia synthesis catalyst into the proton conducting cell has been pursued. These previous studies have generally achieved ammonia production rates on the order of ~10^−12^–10^−9^ mol cm^−2^ s^−1^ under ambient conditions, with champion studies reaching low 10^−8^ mol cm^−2^ s^−1^ production rates^31–33^. Furthermore, most prior studies have used H_2_ rather than H_2_O as the starting feedstock, and H_2_-to-NH_3_ conversion rates, when noted, are generally <0.1%. In contrast, modern Haber-Bosch (HB) plants reach 18% H_2_ single-pass conversion and the conversion efficiency can reach ~98% through H_2_ recycling^34^. Therefore, while direct electrochemical ammonia synthesis is still a hopeful prospect, a two-stage strategy that enables independent optimization of H_2_ production rate in an electrochemical cell followed by a moderate-pressure Haber-Bosch type ammonia synthesis loop may be a more viable intermediate solution. While the results presented in this study focus principally on ambient-pressure operation with promising ammonia production rates exceeding 10^−8^ mol cm^−2^ s^−1^, as a proof of concept and shown in latter section we have also operated our ammonia synthesis loop at pressures up to 12.5 bar, yielding ammonia production rates exceeding 1 × 10^−6^ mols NH_3_ cm^−2^ s^−1^, underscoring the promise of this approach.

### A reversible catalyst for NH_3_ cracking and production

Our reversible approach leverages a novel, recently developed ammonia synthesis catalyst based on a ruthenium-decorated (1–5 wt%) complex multi-cation oxide support, (BaO)_2_(CaO)(Al_2_O_3_); detailed characterization including crystal structure analysis, packed-bed ammonia synthesis kinetics models, and machine learning insights into this novel catalyst have been documented previously^35^. Here we focus primarily on coupling this catalyst to our RePCFCs for both ammonia production and power generation. In order to facilitate mass transport and minimize pressure change in the ammonia synthesis/cracking reactor when coupling to a ceramic cell, we mixed the Ru-B2CA with insulation fibers as shown in Fig. 1b. EDX elemental mapping of as-synthesized Ru-B2CA catalyst powder shows broad compositional uniformity (Fig. 1c), and indicates a Ru and B2CA core-shell structure with a thin and remarkably homogeneous Ru coating layer. The thin Ru coating layer leads to high dispersion, effectively increasing the active Ru surface sites per mass of Ru metal and contributing to the high catalytic activity. Additional characterization is provided in Fig. S1 in the Supplementary Information.

While previous high flow-rate, high pressure packed-bed reactor experiments identify a clear peak in Ru-B2CA NH_3_ synthesis performance at ~490 °C^35^, the ambient pressure, low flow-rate reactor we developed to integrate with the RePCFC in the present work leads to a broader, lower-temperature peak in performance from ~350–450 °C (Fig. 1e). Notably, this temperature window is lower than the typical working temperature of conventional iron-based HB catalysts (450–600 °C)^36,37^ and enables effective NH_3_ synthesis under milder-than-typical HB conditions. Further catalyst characterization is shown in Fig. 1f, where exhaust NH_3_ mole fraction is measured over a range of H_2_: N_2_ ratios at 400 °C. Peak NH_3_ conversion is obtained at ~2.7 H_2_: 1 N_2_. This is slightly shifted from the stochiometric 3 H_2_: 1 N_2_ ratio, suggesting a possible modest impact from H_2_ poisoning at this low synthesis temperature. Prior catalytic characterization of the Ru-B2CA system shows that H_2_ poisoning is eliminated at higher synthesis temperatures (e.g., 490 °C) with a broad maximum in NH_3_ synthesis performance from ~3 H_2_: 1 N_2_ all the way up to 6 H_2_: 1 N_2_ ratio, thereby providing a wide operating window for optimizing performance._._ Measured NH_3_ conversion closely tracks equilibrium predictions above 400 °C, suggesting thermodynamics rather than kinetics limit this catalyst system at these temperatures and flow rates.

Finally, the cracking efficiency of the Ru-B2CA catalyst is shown in Fig. 1d. Cracking efficiency reaches ~100% above 500 °C. Although trace NH_3_ likely remains after cracking, the RePCFC is tolerant to trace NH_3_ without loss in performance or durability. This is in contrast to low-temperature PEMFC-based systems, which are generally intolerant to trace NH_3_ and require further fuel-stream purification prior to use.

### Protonic ceramic fuel cell characterization

**Fig. 2:**
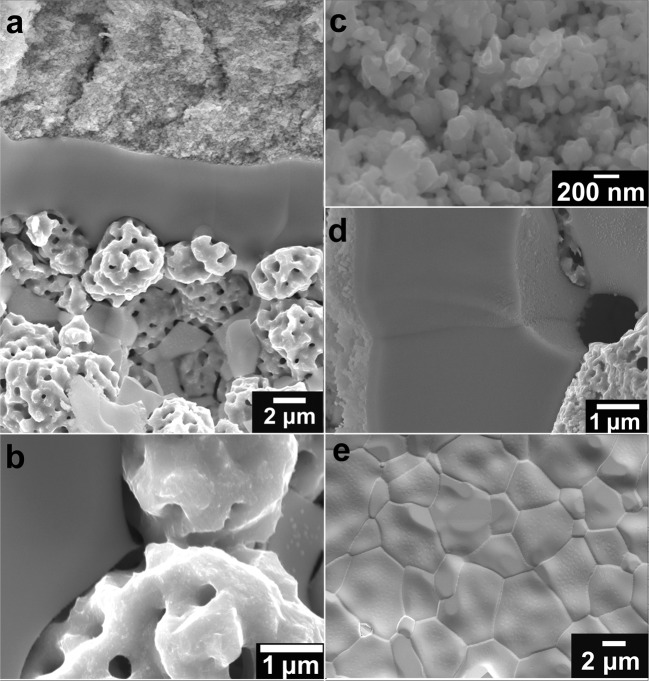
SEM images of proton-conducting electrochemical cell with slurry coated electrolyte layer. **a** Representative cross-sectional SEM of the RePCEC “button” cell comprised of a ~500 μm thick porous Ni-BCZYYb (BaCe_0.7_Zr_0.1_Y_0.1_Yb_0.1_O_3-δ_) cermet electrode support with a ~4 μm-thick dense BCZYYbN electrolyte (BaCe_0.7_Zr_0.1_Y_0.1_Yb_0.1_Ni_0.04_O_3-δ_) and a ~20 μm-thick porous BCFZY (BaCo_0.4_Fe_0.4_Zr_0.1_Y_0.1_O_3-δ_) triple-conducting oxide cathode. **b** High magnification of negatrode showing proton conducting phase (BCZYYb) and electron conducting phase (Ni, with nano-pores), **c** High magnification of positrode showing nano-sized catalyst. **d** High magnification of electrolyte showing visible grain boundary between single grains. **e** Surface view of grains in the electrolyte.

Figure 2 provides representative cross-sectional and surface-view scanning-electron micrograph (SEM) images of our PCFC button cell. The button cell is centered on a 4-μm-thick, dense electrolyte of BaCe_0.7_Zr_0.1_Y_0.1_Yb_0.1_Ni_0.04_O_3-δ_ (BCZYYbN). This electrolyte is mechanically supported on a ~500-μm thick porous composite Ni- BaCe_0.7_Zr_0.1_Y_0.1_Yb_0.1_O_3-δ_ (BCZYYb) cermet fuel electrode (negatrode), with a ~20-μm-thick porous BaCo_0.4_Fe_0.4_Zr_0.1_Y_0.1_O_3-δ_ (BCFZY) triple-conducting oxide air-steam electrode (positrode). SEM images also reveal a “bamboo-like” grain structure for the dense electrolyte layer, so that single BCZYYbN grains separate the porous electrodes without intervening grain boundaries, thereby minimizing ohmic resistance. While BCZYYb synthesized by traditional solid-state reaction methods using 1 wt% NiO as a sintering aid leads to a small but distinguishable second phase impurity, sol-gel synthesized BCZYYbN yields a high-purity single phase material (Fig. S2), and thus is used for RePCFC electrolyte fabrication in this work. A wet slurry coating process is used to form the thin and dense proton-conducting electrolyte layer on the porous negatrode support. Further details on synthesis and cell fabrication are provided in the “Methods” section, while key steps of the cell fabrication process and cell testing are summarized in Fig. S3.

### Fuel cell performance

**Fig. 3:**
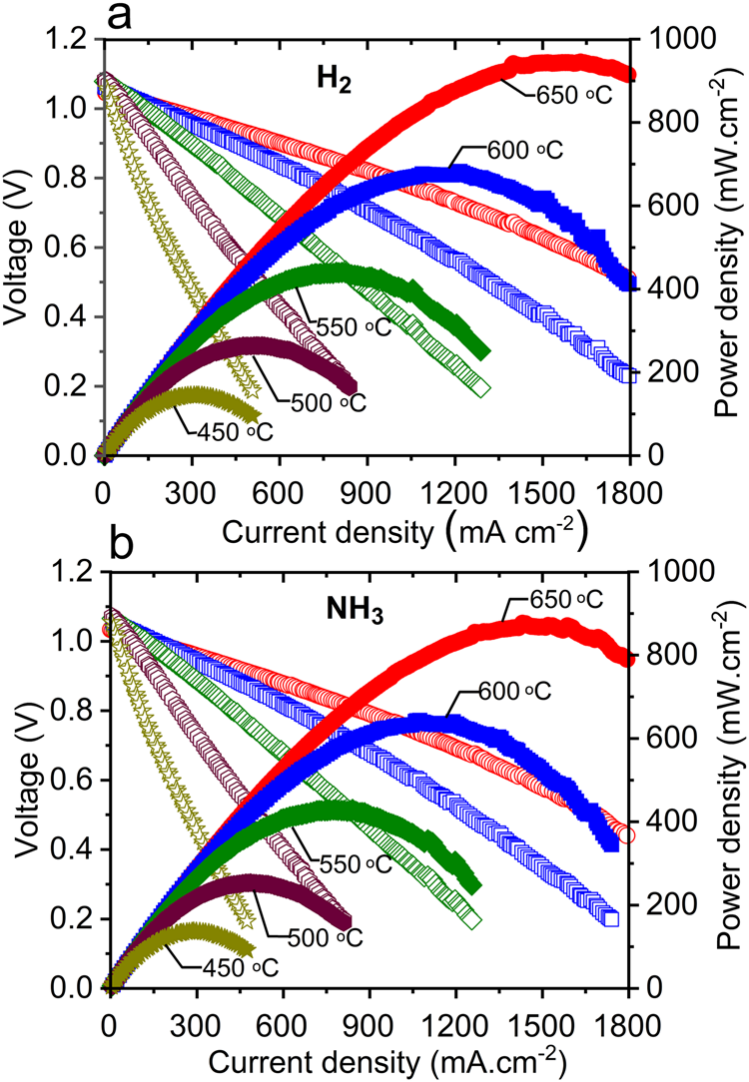
Fuel cell performance under H_2_ and NH_3_ fuels. **a** Fuel-cell polarization under dry H_2_ fuel; 3% humidified air was used as oxidant gas. **b** Fuel-cell polarization curves under dry cracked NH_3_ fuel. Ammonia cracking temperature was 650 °C. The 3% humidified air was used as oxidant gas.

The cells with dense and thin BCZYYbN electrolyte layer were first tested under H_2_ fuel as a baseline reference. Figure 3a shows electrochemical performance under fuel-cell/power-generation mode, with maximum power density (MPD) varying from 944 to 145 mW cm^−2^ as temperature varies from 650 to 450 °C. These are among the highest performances reported for a PCFC in this temperature range^3–7^.

Figure 3b provides the corresponding performance for the same cell when supplied with neat NH_3_ fuel, which is first cracked by passing through the Ru-B2CA catalyst bed. MPD varies from 877 to 137 mW cm^−2^ as the temperature is varied from 650 to 450 °C, representing 93–98% of the performance under H_2_ fuel. This comparison suggests that the Ru-B2CA provides highly effective NH_3_ cracking; we attribute the minor decrease in performance to the dilution of H_2_ in the cracked NH_3_ fuel relative to a pure H_2_ feed stream. Although trace NH_3_ likely remains in the cracked fuel stream, as we will discuss later, the residual NH_3_ does not appear to affect the long-term stability of the cell. Representative electrochemical impedance spectroscopy (EIS) results are shown in Fig. S4. Supplementary Table S1 provides a review of fuel-cell performance under NH_3_ fuel; our performance is among the highest demonstrated.

### Degradation mechanism in NH_3_-fed PCFC

**Fig. 4:**
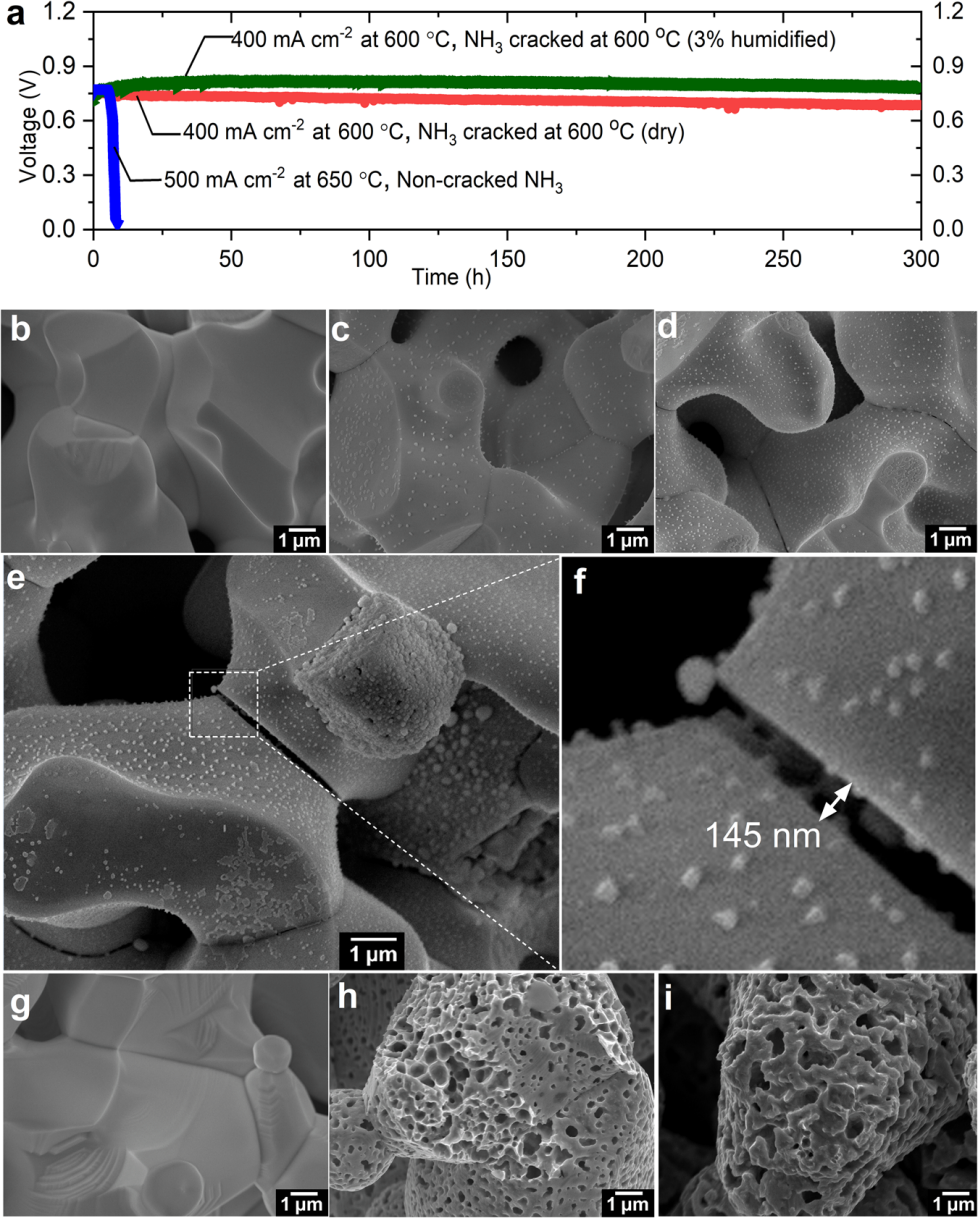
Investigation on stability and degradation mechanism in NH_3_-fed PCFC. **a** Stability test for NH3-fed PCFC with and without Ru-B2CA catalyst. **b**, **c** SEM images of as sintered BCZYYb (with 1 wt% NiO added via solid state reaction synthesis process) and after H_2_ reduction, respectively. **d**–**f**, BCZYYb (with 1 wt% NiO) after non-cracked NH_3_ reduction at different magnification and grains boundaries. **g**–**i** SEM images of as sintered NiO, after H_2_ reduction and after non-cracked NH_3_ reduction, respectively.

In general, we find cells that are directly supplied with NH_3_ without first cracking the fuel show fast degradation with the voltage dropping precipitously within a couple hours to days under operation depending on variations in cell fabrication and testing. Figure 4a shows a representative cell fabricated using the method reported in previous study by Duan et al.^3,6^, the cell initially had high performance, however the voltage dropped to zero after ~15 h of direct NH_3_ exposure. Degradation rates are significantly suppressed in cells using Ru-B2CA as an upstream NH_3_-cracking catalyst. We examined long-term stability by varying the current density and the humidity in the fuel and oxidant streams while maintaining the same temperature for both PCFC operation and NH_3_ cracking. Representative results from cracked NH_3_-fed cells are also shown in Fig. 4a. Degradation rates are significantly decreased relative to the neat NH_3_-fed cells. In order to rule out effects from cell fabrication and testing procedures, a cell was tested up to 1250 h under a variety of testing conditions (Fig. S5). The results show the degradation is more sensitive to operation conditions such as discharge current density and humidity, rather than trace NH_3_ in the fuel, i.e., the intrinsic degradation nature of the PCFC operating under cracked NH_3_ fuel is similar to one operating under neat H_2_ fuel.

For cells supplied with neat (uncracked) NH_3_, we found that both the ohmic and polarization resistances increase dramatically (e.g., by 83x and 50x, respectively in Fig. S6a) as the cell voltage drops to zero. Interestingly as also shown in Fig. S6a, the voltage drop in the cell can be well fitted by an exponential decay function, which indicates a slow yet continuous and time-dependent internal failure occurring therein. Postmortem investigation across several areas of the cell did not reveal any obvious microstructural changes or cracks in the electrolyte that could be responsible for the precipitous decrease in performance and OCV (Fig. S6b).

In order to further understand the causes for the rapid drop in performance and changes in cell resistance under neat NH_3_ fuel, we designed an experiment to isolate the two components in the fuel electrode: the electronic conducting phase (Ni) and the ionic conducting phase (BCZYYb). We prepared both porous NiO and porous BCZYYb (with 1 wt% NiO as sintering agent) pellets and heat treated the samples firstly in H_2_ then in NH_3_.

**Fig. 5:**
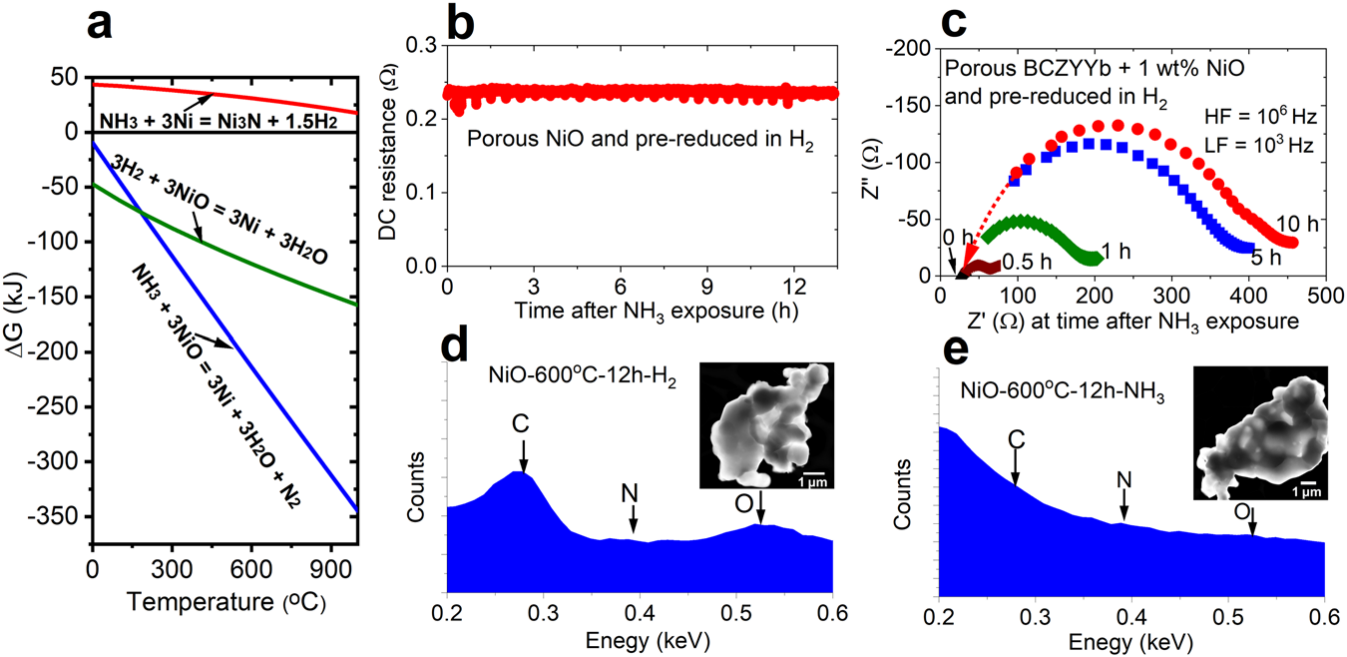
Thermodynamic properties, electrochemical measurements and micro-characterization of degradation mechanism in NH_3_-fed PCFC. **a** Thermodynamic calculations for NiO reduction by H_2_ and NH_3_, and Ni_3_N formation via NH_3_ gas and metallic Ni. The figure shows that NH_3_ is a stronger reducing agent than H_2_ at temperatures exceeding 275 °C, and that Ni_3_N formation is thermodynamically unfavorable under typical PCFC operation temperatures. **b** DC resistance measurement of porous nickel pellet upon non-cracked NH_3_ exposure at 600 °C; steady performance is observed. **c** EIS measurement of a porous BCZYYb (with 1 wt% NiO) pellet during exposure to non-cracked NH_3_ at 600 °C; polarization resistance increases by orders of magnitude over 10 h. **d**, **e** TEM-EDS analysis of nano-sized NiO after H_2_ and non-cracked NH_3_ exposure respectively. The comparison does not show the formation of Ni_3_N.

SEM images of as-sintered BCZYYb pellets after H_2_ reduction or after NH_3_ reduction are shown in Fig. 4b–d. The results indicate a much larger population of nano-sized nickel particles precipitated on the NH_3_-treated BCZYYb sample than the sample treated only in H_2_. We also found a significant number of micro gaps along the grain boundaries of the BCZYYb phase after NH_3_ exposure. The differences are best observed in the larger viewing field images (Figs. S7–S9) or at higher magnification as shown in Fig. 4e and f, where a nano-sized gap between two adjacent grains is clearly seen in the NH_3_-treated sample, with visible exsolution of Ni particles inside the gap as well. We note that these grain boundary gaps also appear in the H_2_-treated sample; however, the gap widths and overall gap density are greatly reduced compared to the NH_3_-treated sample. Similarly, NH_3_-treatment of the NiO sample results in a more highly porous, heavily reduced Ni electrode morphology than seen with the H_2_-treated NiO sample, (Fig. 4g–i, and Figs. S10–S12). While the kinetics of NiO reduction reactions will impact morphological corrosion, the more-pronounced damage to the NH_3_-exposed sample is consistent with the thermodynamics of Gibbs free energy shown in Fig. 5a. Ammonia is a more-favorable reducing agent than hydrogen at the 600 °C target operating temperature, potentially leading to damage of the nickel phase of the cermet fuel electrode, and device degradation.

Electrochemical tests were also performed on the porous pellets. Over the testing period, the DC resistance of a nickel pellet, originally pre-reduced in H_2_, remains constant upon subsequent NH_3_ exposure, as seen in Fig. 5b.

In contrast, EIS testing of the BCZYYb pellet reveals a significant increase in resistance during treatment in NH_3_, with the grain boundary arc in the EIS spectra increasing dramatically over the course of 10 h NH_3_ exposure as seen in Fig. 5c. The designation of the arc to grain boundary resistance is based on the characteristic frequency of the response and can be compared to that of the cell before reduction (Fig. S13). The increased grain boundary resistance measured by EIS agrees well with the appearance of the grain boundary gaps as previously imaged in the porous BCZYYb pellets.

Previous studies of NH_3_-fueled SOFCs have attributed rapid degradation and loss in performance to the formation of nickel nitride (e.g., in NH_3_-exposed Ni/YSZ SOFC anodes). In order to investigate this possibility, we performed TEM-based elemental and phase analysis of our NiO pellets after H_2_ and NH_3_ heat treatment. However, we were not able to find any evidence of nickel nitride formation in the NH_3_-treated pellet under our testing conditions, as shown in Fig. 5d and e. Furthermore, we examined NiO - BaZr_0.8_Y_0.2_O_3-δ_ (BZY20) anode powder treated with NH_3_ using XRD. The XRD patterns also did not show signs of Ni_3_N formation in the anode after exposure to NH_3_ gas (see Fig. S14). Thermodynamic calculation indicates formation of Ni_3_N is highly unfavorable and therefore unlikely as a predominant phase at the testing temperatures as seen in Fig. 5a. Note this does not exclude possible trace amounts of Ni_3_N at the surface dictated by thermodynamic equilibrium. Therefore, we assert that the lower operating temperatures of PCFCs likely lead to different degradation mechanisms vs. NH_3_-fed SOFCs. Overall, our results suggest that at operating temperatures of about 600 °C, the BCZYYb electrolyte phase in the anode support (rather than the nickel phase) is mostly responsible for the fast PCFC degradation rate when operating under NH_3_ fuel without ammonia cracking catalyst.

### Ammonia production and reversible operation

**Fig. 6:**
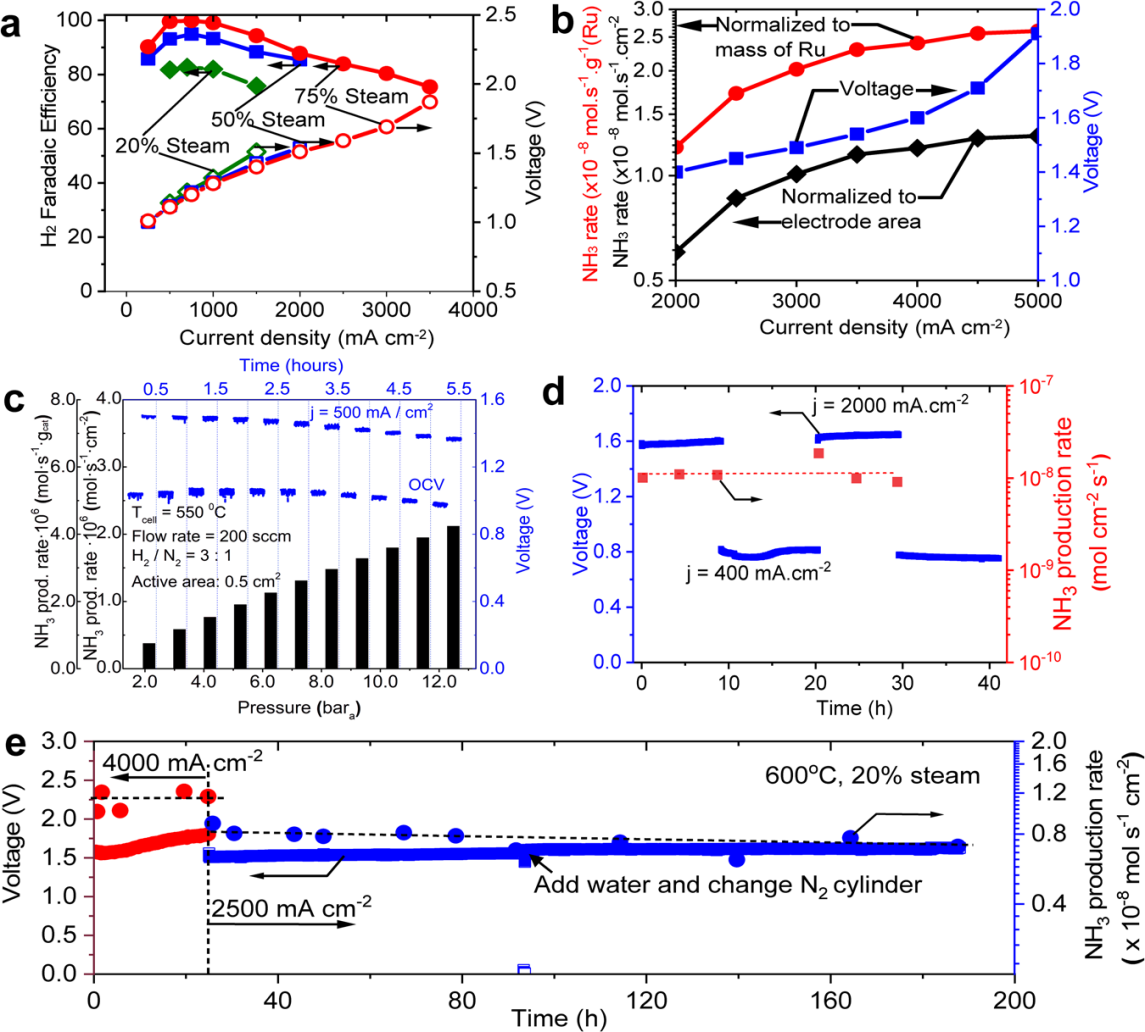
Ammonia production and reversible operation. **a** Measured FE and voltage as a function of applied current density at 600 °C. For FE measurement, ultra-high purity N_2_ was used as carrier gas. **b** Cell potential and NH_3_-synthesis rate as a function of current density at ambient pressure (NH_3_ synthesis rate is shown normalized to both cell active area and catalyst mass). PCFC at 600 °C and ammonia synthesis at 400 °C. **c** NH_3_-synthesis rate as a function of operating pressure from 2 to 12.5 bar_a_. NH_3_-synthesis rate (on the primary y-axis) is normalized both by catalyst loading (g_cat_) and electrochemically active area (0.5 cm^2^). During the nearly 6 h of NH_3_ synthesis, the electrochemical cell was driven at a current density of 0.5 A cm^−2^, while additional H_2_ was supplied to the reactor to maintain 3:1 H_2_:N_2_ stoichiometry. Open-circuit voltage and cell potential during discharge are shown on the secondary y-axis. Driving voltage is found to decrease with increasing operating pressure. **d** Reversible NH_3_-synthesis/NH_3_ fuel-cell operation with two 20-hour cycles. PCFC cell at 600 °C, ammonia synthesis at 400 °C and ammonia cracking at 600 °C. **e** Stability under ammonia production modes for over 160 h of continuous operation. PCFC at 600 °C, ammonia synthesis at 400 °C.

Electrolysis and ammonia production performances from a ~4 µm thick BCZYYb electrolyte-based RePCEC are shown in Fig. 6a, b, where maximum current density reaches to 5000 mA cm^−2^. Gas chromatography (GC) shows nearly 100% H_2_ FE between 500 and 1000 mA.cm^−2^ under 75% steam concentration, greater than 90% FE between 1000 and 1800 mA cm^−2^, and greater than 80% FE between 1800 and 3000 mA cm^−2^. The FE shows nearly linear dependence at current densities greater than 1000 mA cm^−2^.

High electrolysis current density is desired to enable a high NH_3_ synthesis rate. In Fig. 6b, the NH_3_-synthesis rate is shown to increase from ~6 × 10^−9^ to ~1.3 × 10^−8^ mol NH_3_ cm^−2^ s^−1^ as the driving current density is increased from 2000 to 5000 mA cm^−2^, based on an electrochemically active cell area of 0.5 cm^2^. Results normalized to catalyst loading are also shown, ranging from 1.2 × 10^−8^ to 2.7 × 10^−8^ mol NH_3_ s^−1^ g^−1^(Ru). It should be noted that the ammonia production rates per mass of Ru in Fig. 6b are low compared to other Ru-based catalysts^38,39^ for two reasons. First, Fig. 6b employed an ammonia synthesis reactor that was originally built for much larger H_2_ production rates, resulting in a significant excess of catalyst under the testing conditions achieved here. Second, while NH_3_ synthesis is generally maximized at or near the stochiometric ratio of 3:1 H_2_:N_2_, our testing conditions resulted in significant excess N_2_ (e.g., from ~1:6 to 1:70 H_2_:N_2_ depending on the current density), limiting the risk of hydrogen poisoning. These off-stoichiometric conditions greatly affected the production rates using the same catalyst as shown in Fig. 1f. Indeed, when operated under simulated 3:1 H_2_:N_2_ gas in the same reactor, the Ru-B2CA catalyst attained much higher production rates under ambient pressure conditions as shown in Fig. S15. The production rates in Fig. 6b are therefore limited primarily by thermodynamics and H_2_ supply, not catalytic capacity.

The NH_3_-synthesis rate is found to increase sub-linearly with increasing current density, flattening above 3.5 A cm^−2^. We attribute this to the decreasing H_2_ FE with increasing current density under high-current-density operation as shown in Fig. 6a. The NH_3_-synthesis rate tracks linearly with increasing H_2_-flux, taking into account the reduction in H_2_-FE at higher current densities, as shown in Fig. S16.

We note that our ammonia production rates, although they are among the highest values so far reported based on direct steam electrolysis, are still far from practical application. Our demonstration lacks the pressurization and recycling strategies used in a conventional HB ammonia-synthesis loops. To simulate the effects of H_2_ recycling and pressurization, we conducted an experiment where both the electrochemical cell and the NH_3_ synthesis reactor were pressurized up to 12.5 bar_a_ while additional H_2_ (beyond that produced by the electrochemical cell) was co-fed with N_2_ at a 3:1 H_2_:N_2_ stoichiometry (see Fig. S17 for a schematic of the pressurized testing setup). Pressurization increased NH_3_ synthesis rates to as high as 2.1 × 10^−6^ mol NH_3_ cm^−2^ s^−1^, more than 100X higher than our ambient-pressure, sub-stochiometric results (Fig. 6c). This result demonstrates the benefit of elevated pressure to both the electrochemical cell performance and the NH_3_-sythesis rate, and the mechanical robustness of the protonic-ceramic electrochemical cell above ambient-pressure operation. Our pressure tests, along with Norby et al.‘s study at 4 bar pressure^18^ suggest that proton-conducting cells have adequate mechanical strength for higher-than-ambient pressure operation. This demonstration also further underscores the value of the hybrid two-reactor approach, since the implementation of H_2_ recycling and mild pressurization can clearly drive significant improvements in the NH_3_ synthesis rate and conversion. Furthermore, the two-reactor approach enables individual optimization of H_2_ production and NH_3_ synthesis so that both can eventually achieve high production rates and high conversion efficiencies. Such optimization is challenged in a “one-cell” approach as it is difficult to independently control crucial variables such as the H_2_ production rate, H_2_:N_2_ ratio, reactor pressure, GHSV, and catalytic residence time in such systems.

A key feature of our hybrid approach is that it directly enables cyclic operation between NH_3_-fueled electric-power generation and electric power-driven NH_3_ synthesis. This cyclic operation is shown in Fig. 6d. Two complete cycles are shown, each consisting of at least 8 h of continuous operation in both operating modes. Ammonia synthesis is reasonably steady at 1 × 10^−8^ mol NH_3_ cm^−2^ s^−1^ at a driving current of 2000 mA cm^−2^, though some performance degradation is observed. Similarly, under fuel-cell mode, the cell potential is steady at 0.75 V while driving 0.4 A cm^−2^.

Stability under long-term synthesis operation was also studied, with encouraging preliminary results as shown in Fig. 6e. Stable PCEC operation under 1400 mA.cm^−2^ at 600 °C for 600 h has been previously demonstrated by our group^2^. To accelerate the stability test and study high current density electrolysis, we directly applied high current densities to the cell at 600 °C. As shown in Fig. 6e, the cell was first tested under 4000 mA.cm^−2^ for 25 h. Fast degradation was observed but the cell nevertheless survived under this high current density test despite the relatively low steam concentration. The degradation rate was immediately reduced upon decreasing the current density to 2500 mA cm^−2^ over the following ~160 h of testing. The stability increased with decreasing current density, as expected. The applied bias necessary to drive the 2500 mA cm^−2^ of electrolysis current increased by about 8% over 160 h which is promising considering the relatively high current density. The ammonia production rates slightly decreased as the voltage went up but were generally stable.

## Conclusions

A novel ruthenium-based catalyst (Ru-B2CA) with high ammonia cracking and ammonia conversion efficiency under ambient- to low-pressure operation was successfully integrated with a protonic ceramic electrochemical cell to enable reversible NH_3_ fuel cell and NH_3_ synthesis operation using a two-chamber design approach. Under power generation mode with Ru-B2CA as the cracking catalyst, maximum power densities (MPD) of 944 and 877 mW cm^−2^ at 650 °C are achieved for H_2_ and NH_3_ fuel, respectively. Power density in NH_3_ fuel-cell mode exceeds 93% of that under pure-H_2_ fuel. The use of the cracking catalyst significantly reduces the cell degradation rate. When fed with uncracked ammonia, the BCZYYb electrolyte phase in the anode support exhibits grain-boundary delamination driven by nickel exsolution on the BCZYYb electrolyte surface and along grain boundaries in the anode support. Use of the Ru-B2CA cracking catalyst enables stable performance over 1200 h of testing. The results suggest that the residual degradation which persists under cracked ammonia is similar to the intrinsic degradation nature of the PCFC running under H_2_ fuel, and is not due to the trace NH_3_ remaining in the fuel stream. Under electrolysis mode operation, we measured nearly 100% H_2_ Faradaic efficiency between 500 and 1000 mA.cm^−2^ under 75% steam concentration. Enabled by the reversible application of Ru-B2CA as a synthesis catalyst, we achieve >1 × 10^−8^ mol NH_3_ cm^−2^ s^−1^ production rates under ambient pressure operation, and >2 × 10^−6^ mol NH_3_ cm^−2^ s^−1^ at 12.5 bar_a_ pressurized operation when supplementing the electrolysis-derived H_2_ with additional cylinder H_2_ to achieve the target 3:1 H_2_:N_2_ stoichiometry. Furthermore, we use our approach to demonstrate cyclic reversible NH_3_ synthesis and NH_3_-fueled power production using a proton-conducting electrochemical device and achieve >160 h continuous ammonia production using H_2_ derived from steam electrolysis with reasonable stability.

## Methods

### Preparation of coarse BCZYYb (BaCe_0.7_Zr_0.1_Y_0.1_Yb_0.1_O_3-δ_) electrolyte precursor

BCZYYb (BaCe_0.7_Zr_0.1_Y_0.1_Yb_0.1_O_3-δ_) was prepared by ball milling BaCO_3_, CeO_2_, ZrO_2_, Y_2_O_3_, and Yb_2_O_3_ according to the desired stoichiometry with 1wt.% NiO added as sintering agent for 24 h using 12 mm yttria stabilized zirconia grinding media in reagent alcohol (200 Proof). The slurry was dried for 15 h at 80 °C. The collected powder was dry ball billed for another 5 h and finally dried at 200 °C for 1 h. This coarser electrolyte material was used in the cermet negatrode electrode support.

### Preparation of positrode (BCFZY, air-steam electrode) and electrolyte (BCZYYbN) nanosize powder by Sol-Gel process

BCFZY (BaCo_0.4_Fe_0.4_Zr_0.1_Y_0.1_ O_3-δ_) was used as the cathode and was prepared as reported in previous studies^2,40^. The following briefly describes the procedure with notes for synthesis of BCFZY cathode powder in this work. (1) Add citric acid and distilled water to a glass beaker under stirring and without heating. (2) Add EDTA to the solution under stirring. (3) Add chemicals in the order of Ba(NO_3_)_2_, Co(NO_3_)_2_•H_2_O, Fe(NO_3_)_3_•9H_2_O, Y(NO_3_)_3_•6H_2_O, and ZrO(NO_3_)_2_ (35wt.% in dilute nitric acid) under stirring. (4) Add NH_3_.H_2_O slowly in the amount of about 1000 ml NH_3_.H_2_O per mole of citric acid or EDTA. The molar ratio of EDTA: citric acid: total metal ions was set as 1.5: 1.5: 1. (5) Set the hotplate to 250 °C with continuous stirring till the gel forms. (6) Dry the gel at 150 °C for 24 h to form a black porous charcoal-like precursor. (7) Manually crush and slightly grind the solid precursor and ball mill the charcoal with yttria stabilized zirconia balls and isopropanol for 24 h. (8) Dry the black charcoal slurry after ball milling at 90 °C for 12 h. (9) Calcine the powder at 600 °C for 5 h. (10) Wet ball mill the calcined powder with yttria stabilized zirconia balls and isopropanol for 72 h to further reduce the particle size and agglomerates. (11) Finally dry the slurry for 24 h at 90 °C. The cathode powder is now ready for making paste. BCZYYbN (BaCe_0.7_Zr_0.1_Y_0.1_Yb_0.1_Ni_0.04_ O_3-δ_) nanosize powder was prepared in a similar way except it was calcined at 800 °C for 10 h.

### BaCo_0.4_Fe_0.4_Zr_0.1_Y_0.1_ O_3-δ_ positrode paste preparation

The BCFZY paste was prepared using the procedure reported in previous study^2^. Briefly, 4 g of as-prepared BCFZY nanopowder was mixed with 1 g of calcined and ground BCZYYb coarse electrolyte powder (calcined at 1400 °C for 10 h with 1.0 wt. % NiO powder), 0.4 g of 5 wt-% V-006A in alpha terpineol as binder, and 1 g of 20 wt-% Solsperse 28,000 in alpha terpineol as dispersant. The materials were manually mixed using mortar and pestle until a viscous and uniform black paste was formed.

### NiO-BaCe_0.7_Zr_0.1_Y_0.1_Yb_0.1_O_3-δ_ negatrode (fuel-electrode) support preparation

Negatrode green pellets in this study were prepared by die pressing. NiO was mixed with aforementioned BCZYYb coarse raw precursors in a weight ratio of 65:35. PVB (Polyvinyl butyral B-74), ethyl cellulose, and V-006 were added as binders in a weight ratio 2:1:1. PVP (Polyvinypyrrolindone, MW.40000) was added as dispersant. Reagent alcohol (200 Proof) with small amounts of 1-butanol and xylene were used as solvents. The mixture was rolling milled for 18 h. In total, 20 wt.% starch-powder pore former was added to the NiO and BCZYYb, and ball milling was continued for another 1 h. The wet slurry was dried at 100 °C for 3 h. The dried powder was then roller milled again for 0.5 h, and sieved using a 40-mesh screen, then sealed and stored for die pressing. When ready for die pressing, reagent alcohol was lightly sprayed over the dry powder, slightly moistening the powder, and promoting adherence between particles during die pressing. One to two grams of anode powder was placed within a 19–20 mm stainless steel die and axially compressed using 49 kN (~11,000 lbs) of force. These parent materials were formed into a two-phase NiO-BCZYYb composite during co-sintering with the electrolyte.

### Preparation of negatrode-supported membrane-electrode assemblies

Following die pressing of the negatrode supports, the green pellets were pre-sintered at 1200 °C for 2 h. The electrolyte layer was deposited by two wet-coating processes: (I) For depositing an electrolyte layer of less than 5 µm thick, a spin coating process was applied which is also the primary method used in this study. This process takes advantage of centrifugal force due to the high speed spinning so that gas bubbles that otherwise may be trapped in other coating process are removed immediately during the spin process, leading to a very dense electrolyte layer with negligible defects. In this study, 30 deposition layers were needed to obtain a 4-µm thick sintered electrolyte layer, leading to a deposition rate of about 130 nm/layer. Higher deposition rates can be achieved by higher solid loading. Figure S3b shows example cells after depositing the electrolyte layer by the spin coating process. (II) For depositing an electrolyte layer of greater than 10 µm thick, drop coating was applied. The liquid amount was calculated and controlled based on the area of the pellets, solid loading, target electrolyte thickness and density of sintered electrolyte. For an electrolyte suspension with a solid loading of ~18 g solid/100 ml solvent as an example, the calculated liquid volume based on an assumed density of 6 g/cm^3^ (applicable to most electrolyte powder) is about 100 µL for a desired 20 µm thick electrolyte layer using a 19 mm die (measured was about 25 µm) and about 150 µL for a desired 15 µm thick electrolyte layer using 25 mm die (measured was about 13.5 µm). Supplementary Figure S3c shows samples after the drop coating process. After coating, the anode-electrolyte assemblies were sintered at 1450 °C for 18 h. After sintering, cells were manually ground to the desired thickness of ~500 µm, and were generally flat for compression sealing, as shown in Fig. S3d–3f. In this study, one cell has a brush painted electrolyte followed by sintering (Used in Fig. 4a and see the notes therein) using the method described in previous study^6^. Cells without special notes all employed the spin-coated electrolyte layer.

### Fabrication and packaging of complete membrane-electrode assemblies

The BCFZY positrode paste was painted onto the electrolyte layer of the sintered negatrode-electrolyte half cells. The cells were then fired at 900 °C for 5 h. This completes fabrication of the membrane-electrode assembly. For high-temperature electrochemical testing, Au or Pt contact pastes were painted on top of the electrodes, and silver grids of ~1 mm spacing were painted atop the contact pastes. Two silver wires (0.5 mm diameter) were attached at the center of the electrodes and then baked at 250 °C on a hotplate. The cells were then sealed to an alumina tube using Ceramabond (552-VFG) bonding agent (See Fig. S3h). Ultra-high purity H_2_ or NH_3_ were used as fuel, with synthetic air (21% O_2_, 79% N_2_) as oxidant during fuel-cell performance testing. Water vapor was added to the synthetic air stream by flowing the oxidizer through a bubbler with controlled temperature. Planar cell was compression sealed to gas plumbing using Thermiculite 870 gasket (See Supplementary Fig. S3i, Ceramabond was used as additional sealant around the edge of cells to improve sealing). A mechanical needle valve was placed downstream of the H_2_ production reactor to regulate the pressure at the ammonia side of the membrane-electrode assembly, while a back-pressure regulator (Equilibar) controls air/steam pressure.

### H_2_ faradaic efficiency (FE)

H_2_ FE over a range of conditions was measured at the fuel-electrode exhaust under electrolysis operation using a gas chromatograph (GC). The GC (Agilent 3000 A) was calibrated using four different concentration H_2_/N_2_ calibration gases. The exhaust gas exiting from the fuel electrode of the membrane-electrode assembly was directly fed to the GC. The FE of the cell is calculated as the ratio of measured H_2_ produced to the theoretical H_2_ produced according to Faraday’s law.

### Catalyst synthesis

The (BaO)_2_(CaO)(Al_2_O_3_) (B2CA) catalyst support was prepared using solid-state synthesis. Stoichiometric ratios of BaCO_3_, CaCO_3_, and Al_2_O_3_ were mixed with heptane in a planetary ball mill for 3 h, then dried in air at 80 °C. When dry, the mixture was calcined in air at 1300 °C for 6 h. The resulting material was then ball milled in heptane a second time to increase surface area, and then allowed to dry. The ruthenium catalyst was deposited onto the B2CA support through wet impregnation. RuCl_3_ •1H_2_O was first dissolved in 50 mL of acetone. Once dissolved, a 50 mL suspension of B2CA in acetone was added to the Ru solution. The mixture was continuously stirred until acetone evaporation was complete to form the catalyst and support. Ruthenium loading can be varied.

### Ammonia reactor

Ru-B2CA catalyst was either mixed with support of refractory insulation fibers with 1:1 weight ratio or alumina with 1:2 weight ratio as specified for corresponding figures in the main text. For atmospheric-pressure testing, the mixed catalyst-support component was gently loaded into a tubular reactor. A small distance at the two ends of catalyst zone was filled with blank insulation fibers to trap catalyst particles that may escape due to gas flow. The reactor was placed within a small clamshell test furnace.

### Ammonia detection

Ammonia Drager tubes (Fisher Scientific) were used to quantify ammonia production rates due to the relatively low ammonia concentration. We are aware of the proposed isotope measurements for electrochemical ammonia synthesis approach^41,42^. Since we have limited access to isotope measurement capability, and our focus is not on single-step electrochemical ammonia synthesis, of which the synthesis mechanism is mostly important, we did not adopt isotope methods but using other calibration-based method. Briefly, we calibrate a batch of commercially available fresh Drager tubes using ammonia calibration gases containing trace amount of NH_3_ in N_2_ (2% Certified Standard from MESA Specialty Gases & Equipment, no listed NOx as contaminant) with controlled flow rates close to synthesis condition. Since each Drager tube gives distinct color difference within its detecting range (See Fig. S18a), we carefully count the traveling time corresponding to each marker and plot the data. The linear slope (Fig. S18b as an example) is defined as indicator number. The process is repeated for different standard gases to give an average indicate number per distance of ammonia tracer agent, which is then used to measure ammonia concentration other than standard values. The tubes were placed downstream of the ammonia-synthesis reactor. For NH_3_ cracking where high ammonia concentration is expected, a single-zone ammonia gas monitor (Bacharach AGMSZ) or gas chromatograph (Shimadzu GC 2014) equipped with a thermal conductivity detector and a flame ionization detector (TCD and FID) were used to measure ammonia concentration in the reactor exhaust stream

## Supplementary information

Supplementary Information

## Data Availability

The data that support the plots within this paper and other findings of this study are available from the corresponding authors upon reasonable request.
