# Supplementary material for: Ammonia-fed reversible protonic ceramic fuel cells with Ru-based catalyst
Source: Commun Chem. 2021 Aug 17;4:121. doi: 10.1038/s42004-021-00559-2 (PMC9814555; doi:10.1038/s42004-021-00559-2)
Supplement: Supplementary file 1 — Supplementary Information [file 42004_2021_559_MOESM1_ESM.pdf]

Supplementary Information for

Ammonia-fed reversible protonic ceramic fuel cell with Ru-based catalyst

Liangzhu Zhu[1,4]*, Chris Cadigan[2], Chuancheng Duan[3], Jake Huang[1], Liuzhen Bian[1], Long Le[2], Carolina H. Hernandez [2], Victoria Avance[1], Ryan O'Hayre[1]*, Neal P. Sullivan[2]*

[1]Metallurgical and Materials Engineering Department, Colorado School of Mines, Golden, USA, 80401

[2]Mechanical Engineering Department, Colorado School of Mines, Golden, USA, 80401

[3]Chemical Engineering Department, Kansas State University, Manhattan, USA, 66506

[4]Ningbo Institute of Materials Technology & Engineering, Chinese Academy of Sciences, Ningbo, China, 315201

*Correspondence to: *zhuliangzhu@nimte.ac.cn*; *rohayre@mines.edu*; *nsulliva@mines.edu*;

This PDF file includes:

Supplementary Tables S1 and S2

Supplementary Discussion: Challenges on electrochemical ammonia synthesis

Supplementary Figures S1 to S15

Supplementary Table S1. Comparison of H_2- and NH_3-fed SOFC and PCFC performance

Anode	Electrolyte	Cathode	OCV (V)		MPD ($mW.cm^{-2}$)		% of MPD	Tem. (°C)	Ref.
			H_2	NH_3	H_2	NH_3			
Ni-BCZYYb	BCZYYbN (4 μm)	BCFZY	1.05	1.04	944	877	93	650	This work
Ni-BCZYYb	BCZYYbN (4 μm)	BCFZY	1.08	1.06	682	643	94	600	This work
Ni-BCZYYb	BCZYYbN (4 μm)	BCFZY	1.11	1.09	440	430	98	550	This work
Ni-BCZYYb	BCZYYbN (4 μm)	BCFZY	1.13	1.13	264	254	96	500	This work
Ni-BCZYYb	BCZYYbN (4 μm)	BCFZY	1.11	1.11	145	137	94	450	This work
Ni-YSZ	YSZ (4-6 μm) + GDC (2-4 μm)	LSCF	1.12	1.03	140	100	71	600	1
Ni-SDC	SDC (10 μm)	BSCF	0.75	0.77	1872	1190	64	650	2
Ni-SDC	SDC (10 μm)	BSCF	0.79	0.77	1357	434	32	600	2
Ni-SDC	SDC (10 μm)	BSCF	0.82	0.8	748	167	22	550	2
Pd foil (50 μm)	BZCY (1 μm)	LSCF	1.06	0.92	810	580	72	600	3
Pd foil (50 μm)	BZCY (1 μm)	LSCF	1.08	1	490	340	69	550	3
Pd foil (50 μm)	BZCY (1 μm)	LSCF	1.12	1.02	240	210	88	500	3
Pd foil (50 μm)	BZCY (1 μm)	LSCF	1.13	0.9	85	71	84	450	3
Ni-YSZ	YSZ (15 μm)	LSM-YSZ	1.07	1.06	80	70	88	650	4
Ni-BCE	BCGP(1000 μm)	Pt	1	0.92	36	28	78	600	5
Ni-YSZ	YSZ (30 μm)	LSM-YSZ	1.08	1.08	94	86	91	650	6
Ni-CGO	BCGO (30 μm)	BSCFO-CGO	1.11	1	223	200	90	650	7
Ni-CGO	BCGO (30 μm)	BSCFO-CGO	1.14	1.12	172	147	85	600	7
Ni-SDC	SDC (24 μm)	SSC-SDC	0.8	0.78	870	467	54	650	8
Ni-SDC	SDC (24 μm)	SSC-SDC	0.84	0.82	580	295	51	600	8
Ni-SDC	SDC (24 μm)	SSC-SDC	0.88	0.85	300	170	57	550	8
Fe^*/Ni-YSZ	SSZ (15 μm)	SSZ-LSM	1.15	1.14	292	266	91	650	9
Fe^*/Ni-YSZ	SSZ (15 μm)	SSZ-LSM	1.15	1.15	286	279	98	650	9
Ni–BZCY	BCZY (35 μm)	BSCF	~1.0	~0.95	335	225	67	650	10
Ni–BZCY	BCZY (35 μm)	BSCF	~1.0	~0.95	280	190	68	600	10
Ni–BZCY	BCZY (35 μm)	BSCF	~1.0	~0.95	225	130	58	550	10
Ni–BZCY	BCZY (35 μm)	BSCF	~1.0	~0.95	180	65	36	500	10
Ni–BZCY	BCZY (35 μm)	BSCF	~1.1	~1.0	135	25	19	450	10
Ni–SDC	SDC (50 μm)	SSC–SDC	0.87	0.87	192	168	88	600	11
Ni–SDC	SDC (50 μm)	SSC–SDC	0.93	0.9	82	65	79	500	11

Notes: 1) OCV: Open circuit voltage; 2) MPD: Maximum power density; 3) % of MPD: 100 x MPD(NH_3)/ MPD (H_2).
4)YSZ: yttria-stabilized zirconia; 5)GDC: gadolinium-doped ceria (CGO is used by authors in other references);
6)LSCF: $La_{0.6}Sr_{0.4}Co_{0.2}Fe_{0.8}O_{3-\delta}$; 7)SDC: $Ce_{0.8}Sm_{0.2}O_{2-\delta}$; 8)BSCF: $Ba_{0.5}Sr_{0.5}Co_{0.8}Fe_{0.2}O_{3-\delta}$; 9)BCE: $BaCe_{0.85}Eu_{0.15}O_{3-\delta}$;
10)BCGP: $BaCe_{0.8}Gd_{0.15}Pr_{0.05}O_{3-\delta}$; 11)CGO: Same as GDC; 12)BCGO: $BaCe_{0.8}Gd_{0.2}O_{3-\delta}$; 13)BSCFO (Same as conventionally used BSCF);
14) SSC: $Sm_{0.5}Sr_{0.5}CoO_{3-\delta}$; 15)$Fe^*$: 2 wt.% by impregnation $Fe(NO_3)_3.6H_2O$ and calcined at 600 °C.
16) SSZ: $Sc_{0.1}Zr_{0.9}O_{1.95}$; 17) BCZY: $BaZr_{0.1}Ce_{0.7}Y_{0.2}O_{3-\delta}$; 18) SSC: $Sm_{0.5}Sr_{0.5}CoO_{3-\delta}$; 19) BCZYYb: $BaCe_{0.7}Zr_{0.1}Y_{0.1}Yb_{0.1}O_{3-\delta}$
20) BCZYYbN: $BaCe_{0.7}Zr_{0.1}Y_{0.1}Yb_{0.1}Ni_{0.04}O_{3-\delta}$; 21) BCFZY: $BaCo_{0.4}Fe_{0.4}Zr_{0.1}Y_{0.1}O_{3-\delta}$

Supplementary Table S2 Comparison of reported electrochemical ammonia production rate under ambient pressure (T* stands for this work)

Catalyst	Negatrode	Electrolyte	Positrode	Proton Source	C#	NH_3 Rate mol s^{-1} cm^{-2}	Ref.	Catalyst	Negatrode	Electrolyte	Positrode	Proton Source	C#	NH_3 Rate mol s^{-1} cm^{-2}	Ref.
Plasma	Plasma	H2O-sulfuric acid	Pt	H_2O	1	7.20E-7	12	Ag-Pd	Ag-Pd	BCNN	Ag-Pd	H_2	2	1.92E-09	13
Li_3N	Al	LiCl-KCl-CsCl-Li_3N	Ni	H_2	1	3.33E-08	14	Ag-Pd	Ag-Pd	LBGM	Ag-Pd	H_2	2	1.89E-09	15
Li3N	Ni	LiCl-KCl-CsCl-Li_3N	Carbon	H_2O	1	2.00E-08	16	Ag-Pd	Ag-Pd	BCZN	Ag-Pd	H_2	2	1.82E-09	13
**Ru-B2CA**	**Ni-BCZYYb**	**BCZYYbN**	**BCFZY**	**H_2O**	**2**	**1.20E-08**	**T***	Ag-Pd	Ag-Pd	LCZ	Ag-Pd	H_2	2	1.76E-09	17
SFCN	SFCN	Nafion	Ni-SDC	H_2	2	1.13E-08	18	Cu	Cu	BZCY	Ni-BZCY	H_2	2	1.70E-09	19
SSN	SSN	Nafion	Ni-SDC	H_2	2	1.05E-08	20	VN-Fe	VN-Fe	BCZY81	Ni-BCZY	CH_4	2	1.67E-09	21
Ag-Pt	SSN	Nafion	Ni-SDC	H_2	2	1.05E-08	22	Ag-Pd	Ag-Pd	BCNN	Ag-Pd	H_2	2	1.64E-09	13
SSN	SSN	SPSF	Ni-SDC	H_2	2	1.03E-08	20	Ag-Pd	Ag-Pd	BCNN	Ag-Pd	H_2	2	1.43E-09	13
Ag-Pd-SBCN	SBCN	Nafion	Ni-SDC	H_2	2	8.70E-09	23	Ag-Pd	Ag-Pd	BCN18	Ag-Pd	H_2	2	1.42E-09	13
Ag-Pd	Ag-Pd	CSO	Ag-Pd	H_2	2	8.20E-09	24	Pt	Pt-C	Nafion	Pt-C	H_2O	2	1.14E-09	25
Ag-Pd	Ag-Pd	CGO	Ag-Pd	H_2	2	7.70E-09	24	Pt	Pt/C	Nafion	Pt/C	H_2O	2	9.37E-10	26
Ag-Pd	Ag-Pd	CYO	Ag-Pd	H_2	2	7.50E-09	24	LSCrF-CGDC	LSCrF-CGDC	Carbonate	SSCo-CGDC	H_2O	2	4.00E-10	27
Ag-Pd-SBCC	SBCC	Nafion	Ni-SDC	H_2	2	7.30E-09	23	Ag-CMN	Ag-CMN	Carbonnate	Ag-Pd	H_2	2	3.27E-10	28
Ag-Pd	Ag-Pd	CLA	Ag-Pd	H_2	2	7.20E-09	24	Ag-CF	Ag-CF	Carbonnate	Ag-Pd	H_2	2	2.32E-10	29
Ag-Pd-SBCF	SBCF	Nafion	Ni-SDC	H_2	2	7.00E-09	23	Fe3Mo3N	Fe3Mo3N-Ag	Carbonate	Ag-Pd	H_2	2	1.88E-10	30
Ag-Pd	Ag-Pd	Oxide-salt	Ag-Pd	CH_4	2	6.95E-09	31	Ag-Pd	Ag-Pd	CSO	Ag-Pd	H_2	2	1.83E-10	24
Ag-Pd	Ag-Pd	BCGS	Ag-Pd	H2	2	5.82E-09	32	PBFCu	PBFCu	Carbonate-CGO	PBFCu	H_2O	1	1.83E-10	33
LSFCu-SDC	LSFCu-SDC	Carbonate-SDC	Ni-SDC	H_2/H_2O	2	5.39E-09	34	SBFCu	SBFCu	Carbonate-CGO	SBFCu	H_2O	1	1.53E-10	35
Ag-Pd	Ag-Pd	BCS	Ag-Pd	H_2	2	5.23E-09	32	LSCF-CGCa	LSCF-CGCa	Carbonate-CGDC	SSCo-CGDC	H_2O	2	1.50E-10	36
Ag-Pd	Ag-Pd	BCC	Ni-BCGO	H_2	2	4.63E-09	37	PBFCu	PBFCu	Carbonate-CGO	PBFCu	H_2O	1	1.07E-10	33
Pd	Pd	SCY	Pd	H_2	2	4.50E-09	38	LCFN	LCFN	Carbonate-CGO	LCFN	H_2O	1	9.21E-11	39
Ag-Pd	BSCF	BCY15	Ni-BCY	H_2	2	4.10E-09	40	LSCF	LSCF	BZY	LSCF	H_2O	2	8.50E-11	41
Cu	Cu	BZCY	Ni-BZCY	H_2	2	4.10E-09	19	CFO-CGDC	CFO-CGDC	Carbonate-CGDC	SSCo-CGDC	H_2O	2	6.50E-11	42
Ag-Pd	Ag-Pd	BCD	Ag-Pd	H_2	2	3.50E-09	43	Pt/Plasma	Pt-C/Plasma	Nafion	Pt-C	H_2O	2	5.30E-11	44
Ag-Pd	Ag-Pd	BCG	Ag-Pd	H_2	2	3.09E-09	45	LSFCu-CGCa	LSFCu-CGCa	Carbonate	SSC-CGCa	H_2O	2	5.00E-11	34
Ru	Ru	BCZY27	Ni-BCZY	H_2	1	2.86E-09	46	Ag	Ag	BZY	Ag	H_2O	2	4.90E-11	41
Ag-Pd	Ag-Pd	BCC	Ag-Pd	H_2	2	2.69E-09	47	Pt	Pt	GDC	Pt	H_2O	2	3.67E-11	48
Ag-Pd	Ag-Pd	BCZS	Ag-Pd	H_2	2	2.67E-09	49	Ag-Pd	Ag-Pd	BCY10	Pt	H_2	2	2.80E-11	50
Ag-Pd	Ag-Pd	BCY	Ag-Pd	H_2	2	2.60E-09	51	Nano-Iron	Pt	Ionic liquids	Ni	H_2O	1	2.30E-11	52
Ag-Pd	Ag-Pd	LSGM	Ag-Pd	H_2	2	2.37E-09	53	Ru	Ru/C	Nafion	-	H_2O	2	2.12E-11	54
Ag-Pd	Ag-Pd	BCNN	Ag-Pd	H_2	2	2.16E-09	13	Fe	Fe	SZY	Ag	H_2	2	6.20E-12	55
Ag-Pd	Ag-Pd	BCY	Ag-Pd	H_2	2	2.10E-09	56	LSRT40	LSTR-BCYR	BCY10	Pt	H2	2	5.00E-12	50
Ag-Pd	Ag-Pd	LCZ	Ag-Pd	H_2	2	2.00E-09	57	Ru/MgO	Ag	YSZ	Pd	H_2O	2	6.90E-14	58

Notes: C# stands for number of chambers. 1 means all gases from two electrodes are mixed in one chamber; 2 means positrode and negatrode gases are seperated from each chamber.

<u>Challenges on electrochemical ammonia synthesis</u>

In general, there are two reaction mechanisms regarding electrochemical production of ammonia from nitrogen and hydrogen: the dissociative mechanism and the associative mechanism [59-64]. These mechanisms are well reported in literature, but with variable nomenclature. For convenience, the reaction mechanisms are reformatted as following.

I) Dissociative mechanism: In the dissociative mechanism, each N_2 molecule forms two N atoms directly upon adsorption on the surface site. These then capture three protons and three electrons from the reaction interface or the reaction medium.

$$(N \equiv N)_g + 2\overset{*}{S} \xrightarrow{k_1,K_1} 2\overset{N_*}{S} \qquad \text{(R1)}$$

$$2\overset{N_*}{S} + 6H^+ + 6e^- \xrightarrow{k_2,K_2} 2\overset{(H\overset{H}{N}H)_*}{S} \qquad \text{(R2)}$$

$$2\overset{(H\overset{H}{N}H)_*}{S} \xrightarrow{k_3,K_3} 2\overset{*}{S} + 2NH_{3,g} \qquad \text{(R3)}$$

II) Associative mechanism

$$(N \equiv N)_g + \overset{*}{S} \xrightarrow{k_4,K_4} \overset{(N\equiv N)_*}{S} \qquad \text{(R4)}$$

$$\overset{(N\equiv N)_*}{S} + H^+ + e^- \xrightarrow{k_5,K_5} \overset{(N\cong NH)_*}{S} \qquad \text{(R5)}$$

$$\overset{(N\cong NH)_*}{S} + H^+ + e^- \xrightarrow{k_6,K_6} \overset{(HN=NH)_*}{S} \qquad \text{(R6)}$$

$$\overset{(HN=NH)_*}{S} + H^+ + e^- \xrightarrow{k_7,K_7} \overset{(HN\simeq\overset{H}{N}H)_*}{S} \qquad \text{(R7)}$$

$$\overset{(HN\simeq\overset{H}{N}H)_*}{S} + H^+ + e^- \xrightarrow{k_8,K_8} \overset{(H\overset{H}{N}-\overset{H}{N}H)_*}{S} \qquad \text{(R8)}$$

$$\overset{(H\overset{H}{N}-\overset{H}{N}H)_*}{S} + H^+ + e^- \xrightarrow{k_9,K_9} \overset{(H\overset{H}{N}\bullet\underset{H}{\overset{H}{N}}H)_*}{S} \qquad \text{(R9)}$$

$$\overset{(H\overset{H}{N}\bullet\underset{H}{\overset{H}{N}}H)_*}{S} \xrightarrow{k_{10},K_{10}} \overset{(H\overset{H}{N})_*}{S} + NH_{3,g} \qquad \text{(R10)}$$

$$\overset{(H\overset{H}{N})_*}{S} + H^+ + e^- \xrightarrow{k_{11},K_{11}} \overset{(H\overset{H}{N}H)_*}{S} \qquad \text{(R11)}$$

$$\overset{(H\overset{H}{N}H)_*}{S} \xrightarrow{k_{12},K_{12}} \overset{*}{S} + NH_{3,g} \qquad \text{(R12)}$$

where $\overset{*}{S}$ in the above reactions is an empty catalyst surface site, $\overset{N*}{S}$ indicates dissociated atomic nitrogen adsorbed on a surface site, $\overset{(N \equiv N)*}{S}$ indicates molecular nitrogen adsorption on surface site, and $\overset{(H\overset{H}{N}H)*}{S}$ indicates molecular NH_3 attached to a surface site. Other intermediate products on surface sites are defined similarly. Rate and equilibrium constants are shown as k_s and K_s, respectively.

The dissociation energy in R1 reaches 945.4 $kJ.mol^{-1}$. Additionally, the electrochemical driving force does no effect [59,61], so that N_2 dissociation is the highly unfavorable rate-determining step.

II) Associative mechanism: The first hydrogenation of nitrogen (R6) is the rate-determining step in the associative mechanism [60,61] [65]. The activation energy is ~ 94.5 $kJ.mol^{-1}$ [60], one order of magnitude lower than that of the rate-determining step in the dissociative mechanism. This makes the associative mechanism more energetically favorable for electrochemical ammonia production.

Following the dissociative mechanism, Singh et al. proposed a model to predict rate of ammonia and hydrogen production based on Langmuir isotherms [61]. The two most-important reactions:

$$r_N \simeq k_N \frac{K_N}{K_H} \tilde{c}_{N_2} \qquad \text{Eq.1}$$

$$r_H \simeq k_H \tilde{c}_{+} \tilde{c}_{-} \qquad \text{Eq.2}$$

where K's are the bulk-to-near-surface equilibrium constants, k's are rate constants, and

$\tilde{c}'s$ are bulk concentration. Note the term $\tilde{c}_{-}$ is not meant to indicate a physical concentration but to account for cases where the electron transfer rate is limiting [61] . The rate-determining step of this model is the first hydrogenation step of N_2 (R5).

The above two equations indicate the rate of ammonia production is zeroth order in the electron and proton concentrations, while the rate of hydrogen production is first order in both electron and proton concentrations. The two equations suggest the ammonia production rate is non-electrochemically driven and the rate is nearly independent of electron and proton concentrations.

In order to increase selectivity, the electron and proton concentrations must be minimized in order to minimize the hydrogen evolution reaction (HER). Detailed strategies on improving ammonia selectivity are proposed Singh et al.[61]. By suppressing the protonic current in proton-conducting cells, the H_2 production rate is reduced, while the nitrogen-reduction rate or ammonia-production rate are nearly unaffected, thereby achieving a higher selectivity to NH_3. This has been observed in a few studies where higher ammonia selectivities were achieved under relatively low current densities [52]. Of course, this approach generally is accompanied by low ammonia production rate due to the low current densities. Simultaneously maintaining high rates of ammonia synthesis and conversion efficiency remains very challenging in practice since the activation barrier of HER is much less than that of NRR. Further, no metal catalyst provides better catalytic selectivity in NRR over HER.

Montoya et al. applied DFT calculations to study electrochemical ammonia synthesis on various metal catalysts [66] . Among the 11 elements explored (Re, Ru Rh, Co, Ni, Ir, Pt, Pd, Cu, Au, Ag),

no catalyst demonstrated a more-negative limiting potential under HER than NRR. Rhenium had the minimum difference between HER and NRR, followed by Ru; both metals are good candidates for high-selectivity ammonia synthesis.

These thermodynamic, kinetic, and catalytic analyses all show that it is fundamentally very challenging to suppress hydrogen-evolution reaction while increasing the nitrogen reduction reaction. Low selectivity to NH_3 is more favorable by nature. While better catalysts with higher selectivity may be found for electrochemical production of ammonia, rethinking the integrated electrochemical H_2 production - ammonia thermocatalytic synthesis coupling may prove more efficient, where higher pressure operation is technically more feasible and unused hydrogen can be recycled back to the NH_3-synthesis reactor.

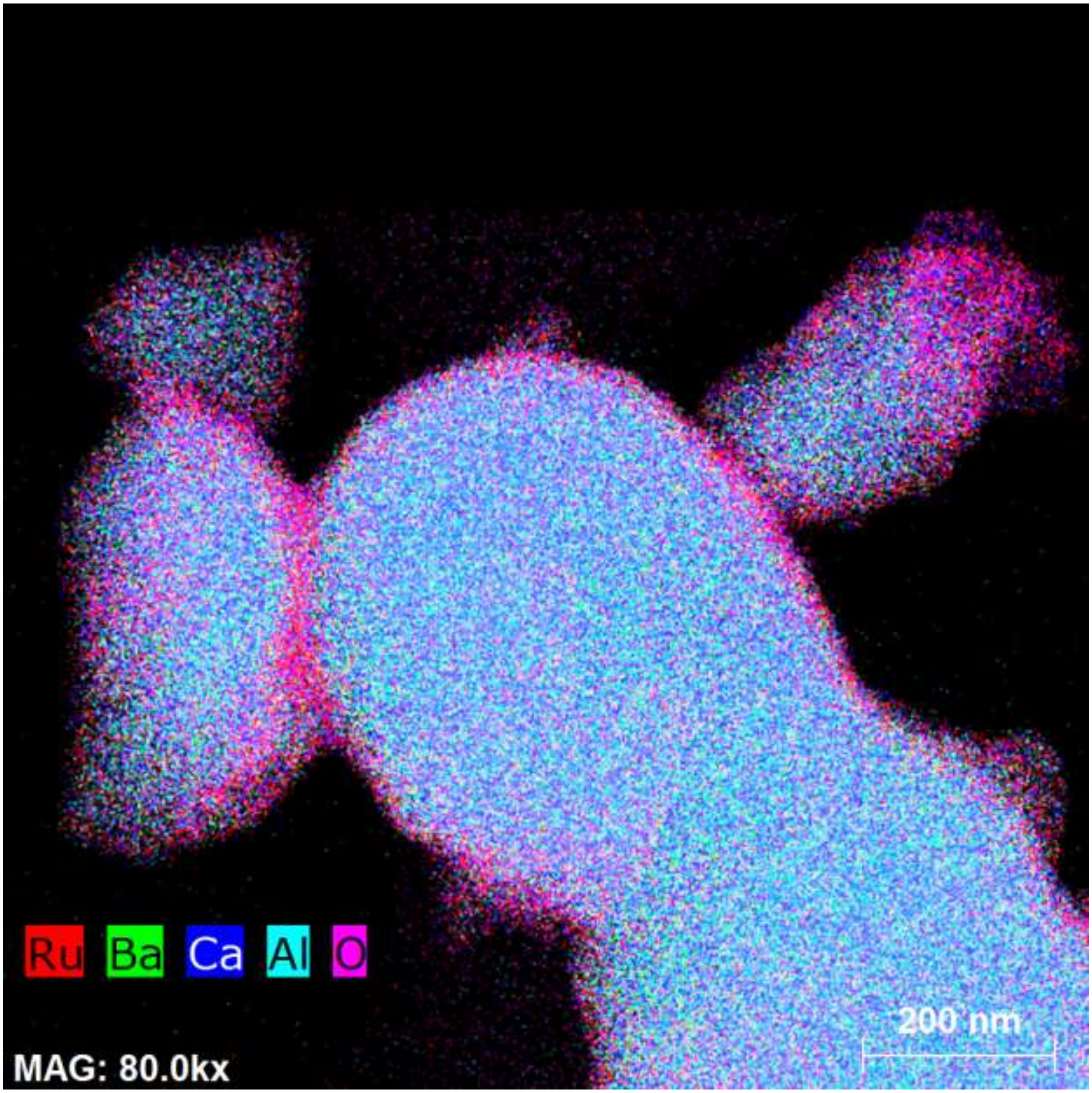

Supplementary Figure S1| TEM-EDX full elemental map showing reasonable uniformity across 300-nm particles and evidence of Ru-B2CA core-shell structure.

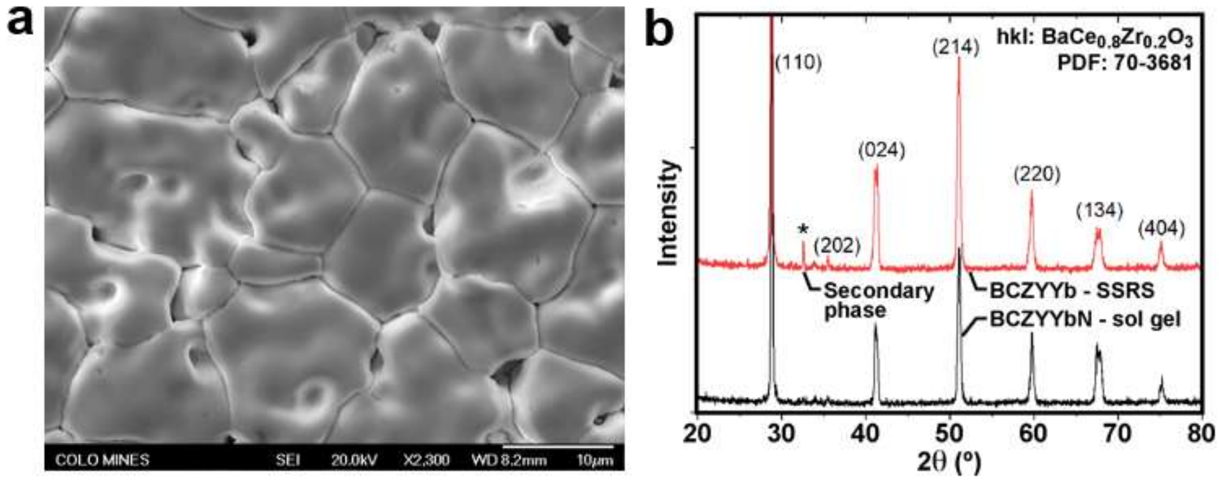

Supplementary Figure S2| Photographs showing. a, SEM image of electrolyte surface via solid state reaction synthesis (SSRS) method, the electrolyte is relatively dense with small defects without affecting cell open circuit voltage or performance. **b**, Comparison of X-ray diffraction spectra of $BaCe_{0.7}Zr_{0.1}Y_{0.1}Yb_{0.1}Ni_{0.04}O_{3-\delta}$ (BCZYYbN) synthesized by sol-gel method and $BaCe_{0.7}Zr_{0.1}Y_{0.1}Yb_{0.1}O_{3-\delta}$ (BCZYYb) synthesized by solid-state reactive sintering (SSRS) (1 wt% NiO was added). Both samples were heat treated at 1450 ^{o}C for 12h. Peaks for both materials are well aligned with database records for $BaCe_{0.8}Zr_{0.2}O_{3-\delta}$. A slight secondary phase is observed in the SSRS material.

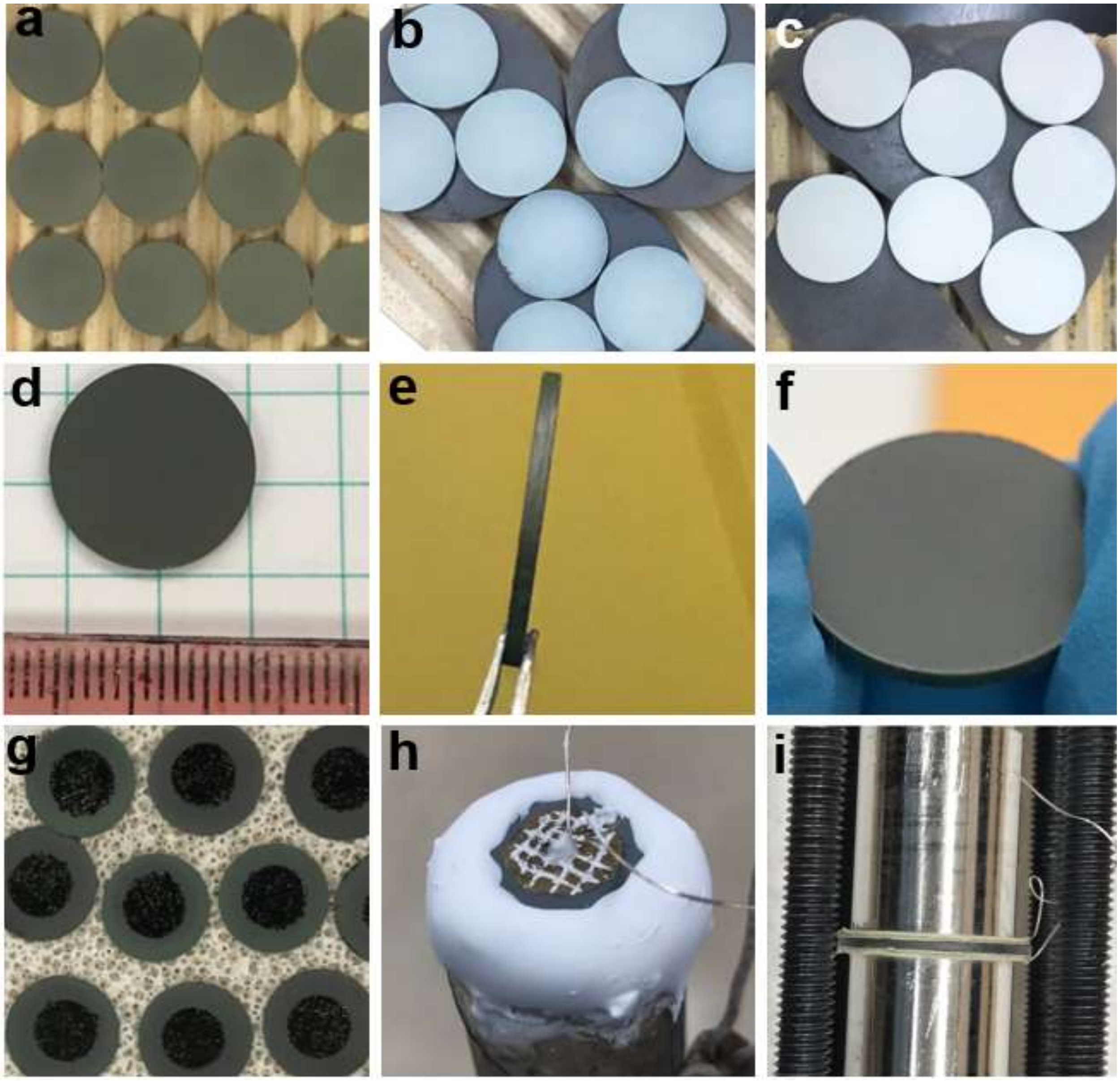

Supplementary Figure S3| Photographs showing. a, Anode green pellets. **b**, Electrolyte layer deposition via spin coating. **c**, Electrolyte layer deposition via drop coating. **d** to **f**, Sintered half-cell. **g**, Cathode application on half-cells. **h**, Example of sealing via ceramabond (552-VFG). **i**, Example of compress sealing.

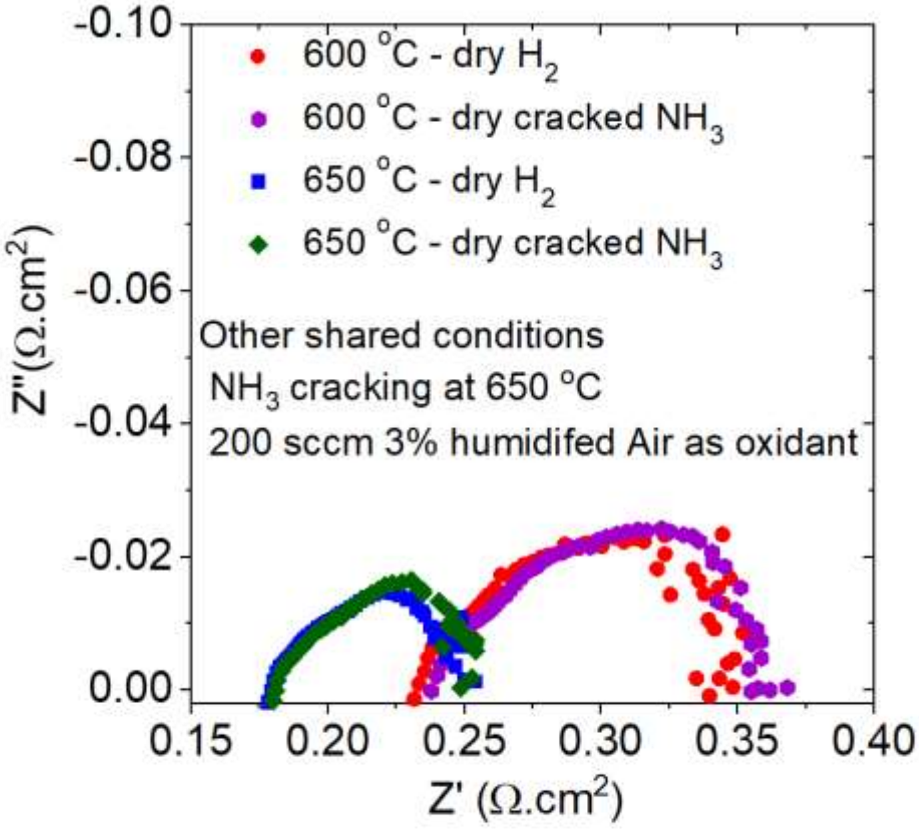

Supplementary Figure S4| Representative EIS at two temperatures under H_2 and cracked NH_3 fuel cell mode.

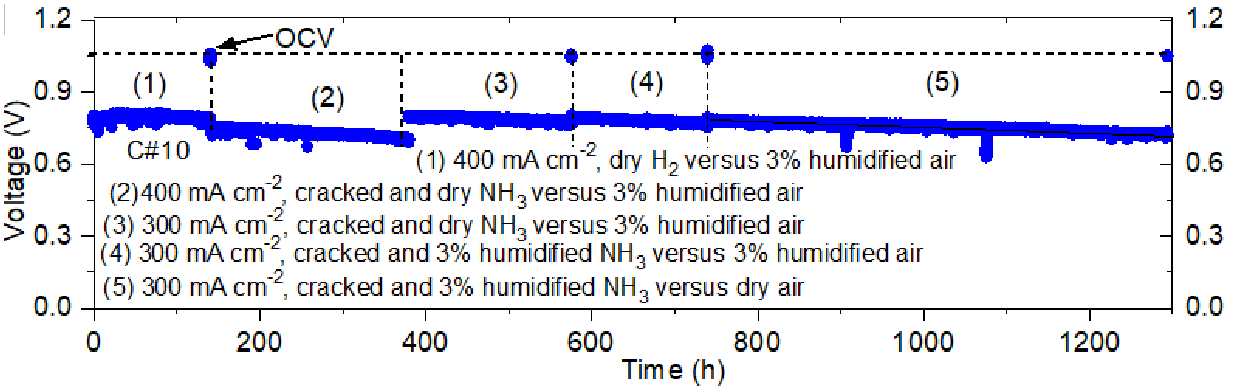

Supplementary Figure S5| Long term stability test of a cell for up to 1250 h under various testing conditions. For cracked NH_3 fuel with trace amount of NH_3, we found the degradation is less sensitive as compared with operation conditions such as discharge current density, humidity, and temperature.

a

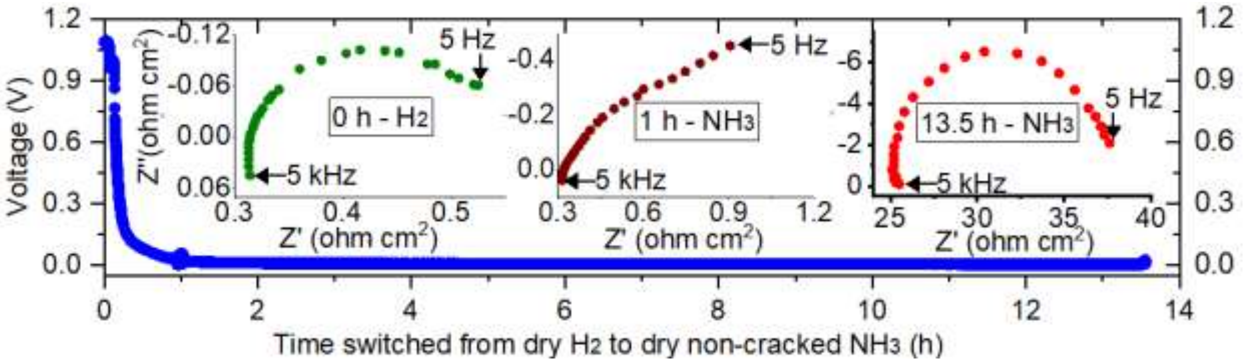

b

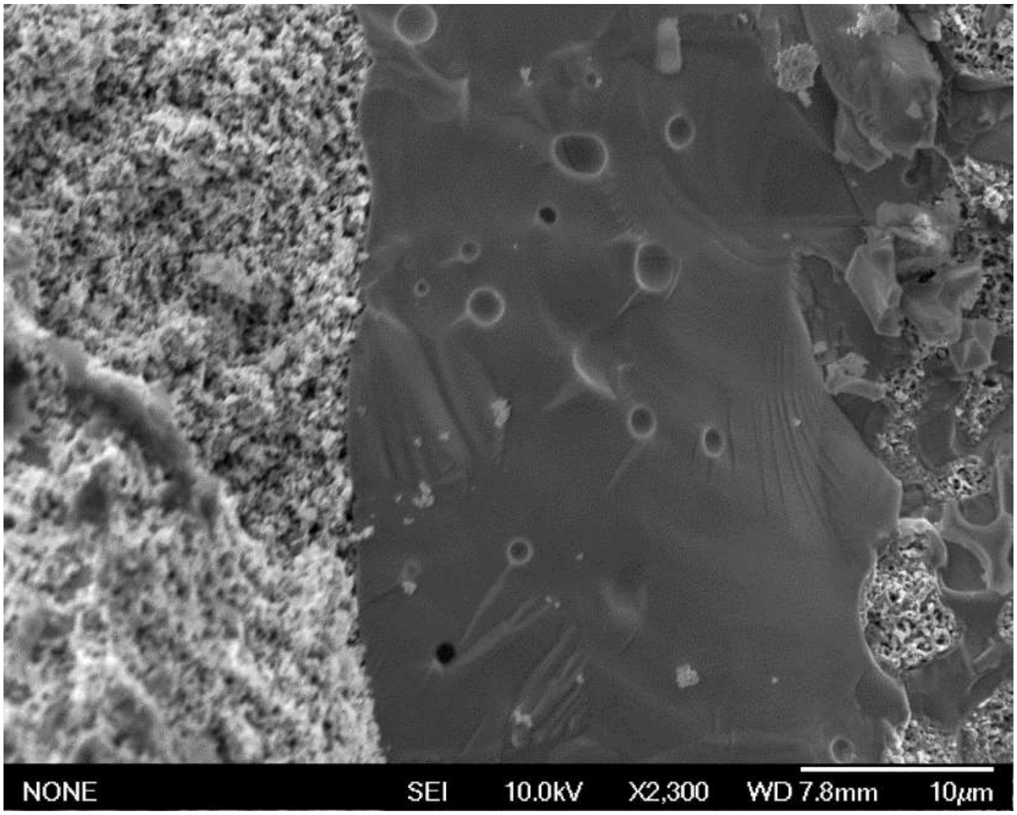

Supplementary Figure S6| Stability test of cell without cracking catalyst and postmortem analysis. a, The cell voltage dropped to zero even under OCV mode after exposure to non-cracked NH_3 fuel. Both the ohmic and polarization resistance increased significantly after cell degraded, respectively by 83 and 50 times higher. **b**, Postmortem analysis shows the cell has a dense and about 25 μm thick electrolyte layer. No mechanic cracks in the electrolyte layer were found based on the measured areas. Note this cell has a drop coated electrolyte layer via SSRS process.

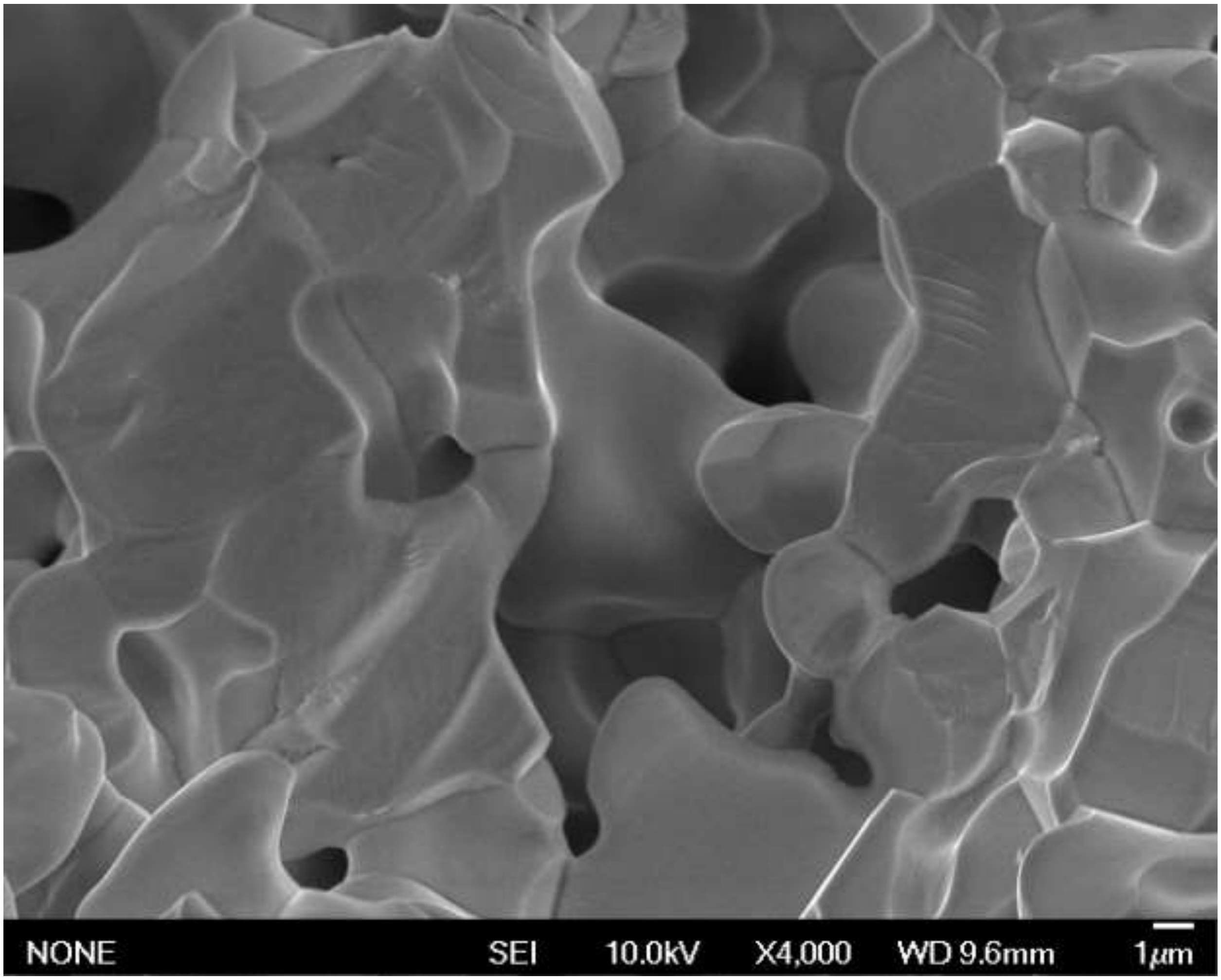

Supplementary Figure S7| Additional and larger viewing field SEM images of assintered porous BCZYYb pellet (with 1 wt.% NiO).

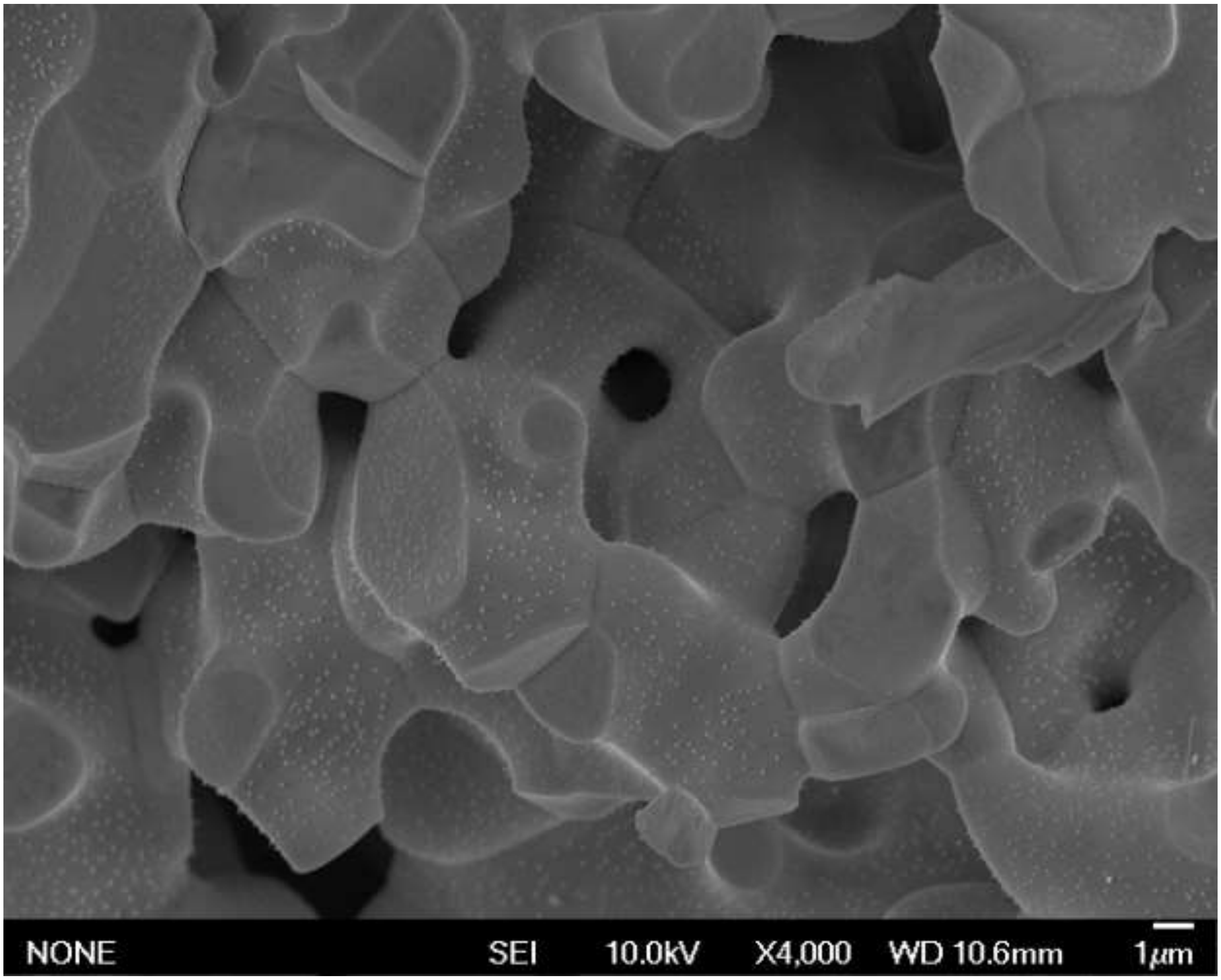

Supplementary Figure S8| Additional and larger viewing field SEM images of porous BCZYYb pellets (with 1 wt.% NiO) after H_2 reduction.

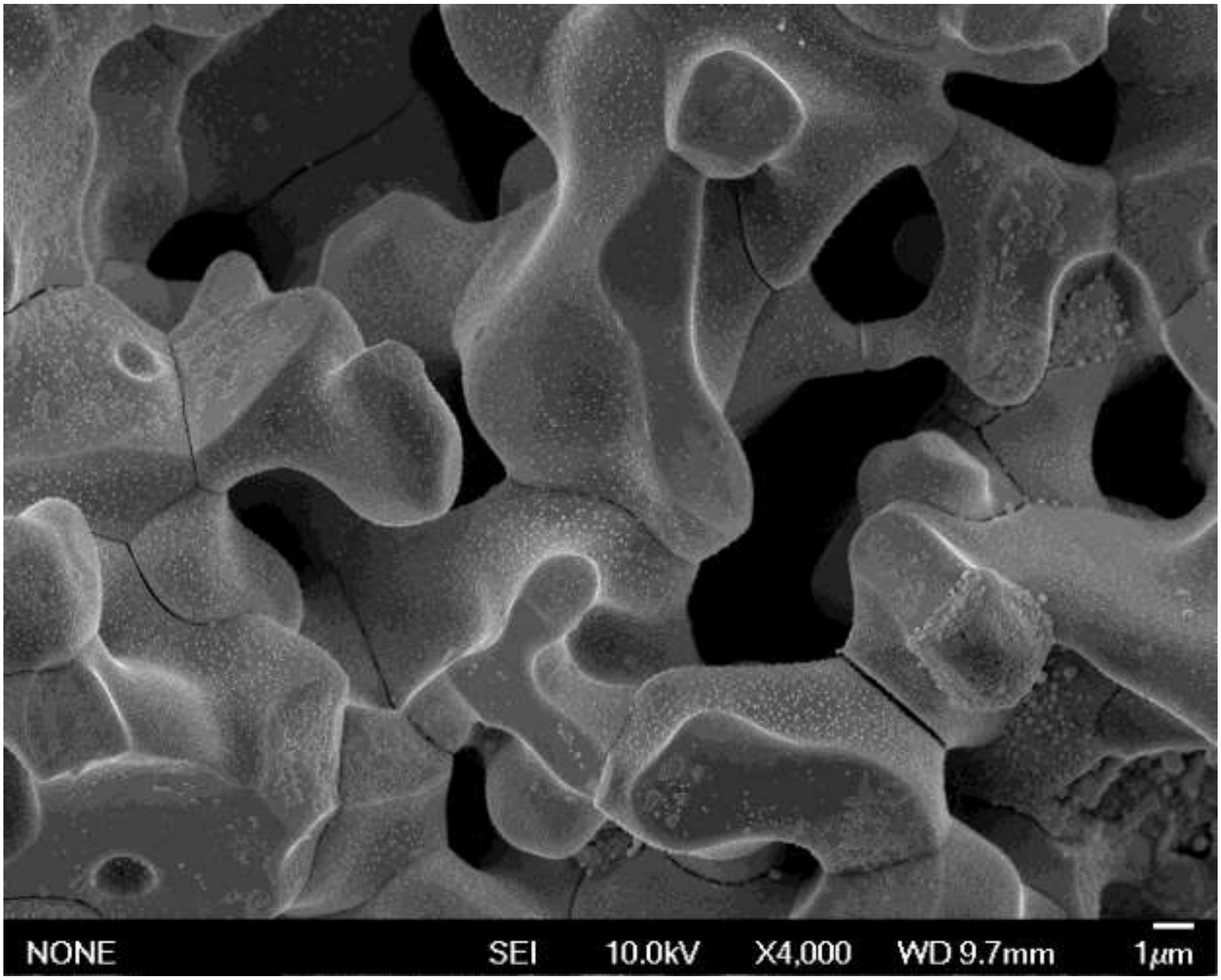

Supplementary Figure S9| Additional and larger viewing field SEM images of porous BCZYYb pellets (with 1 wt.% NiO) after NH_3 reduction.

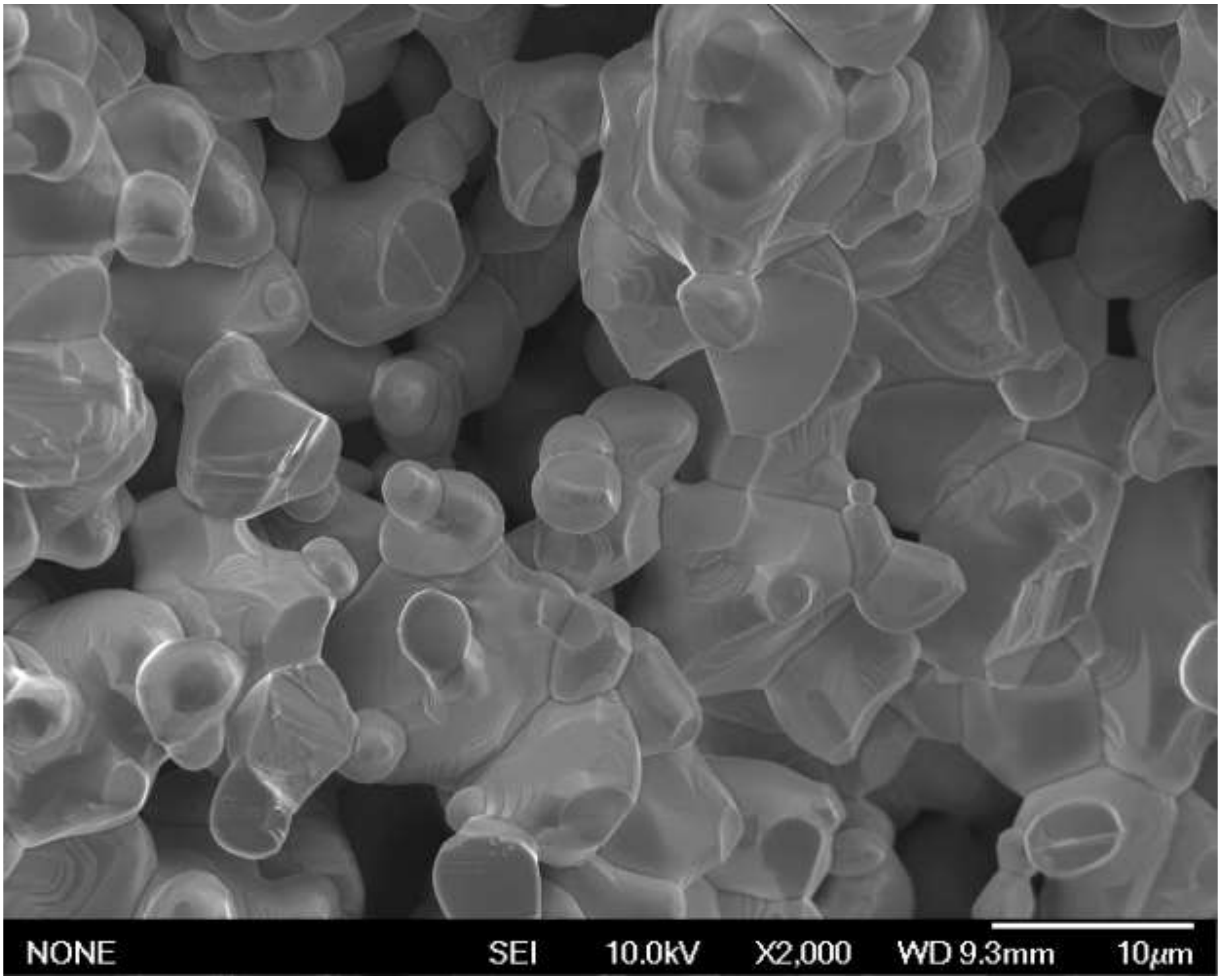

Supplementary Figure S10| Additional and larger viewing field SEM images of as-sintered porous NiO pellet.

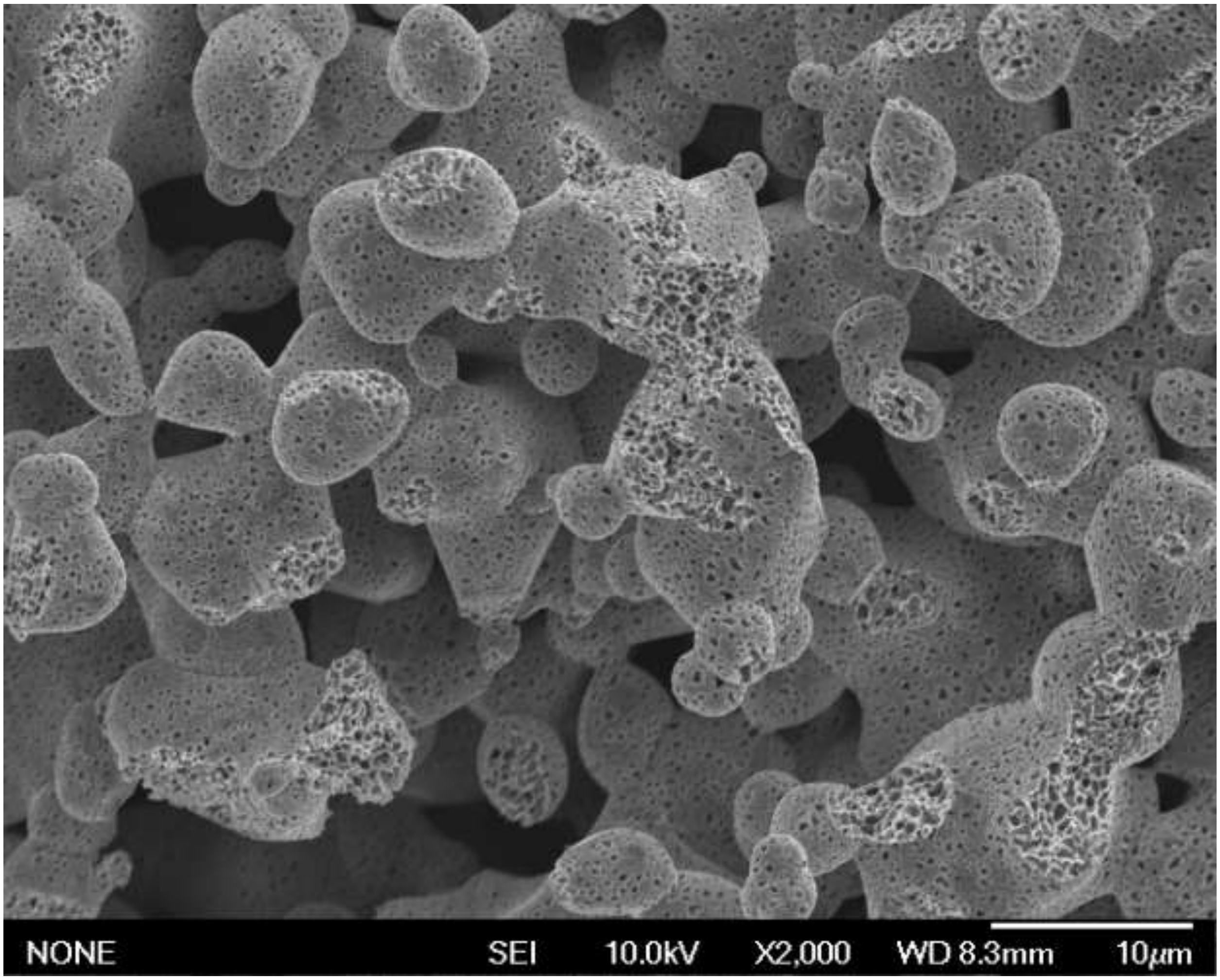

Supplementary Figure S11| Additional and larger viewing field SEM images of porous NiO pellets after H_2 reduction.

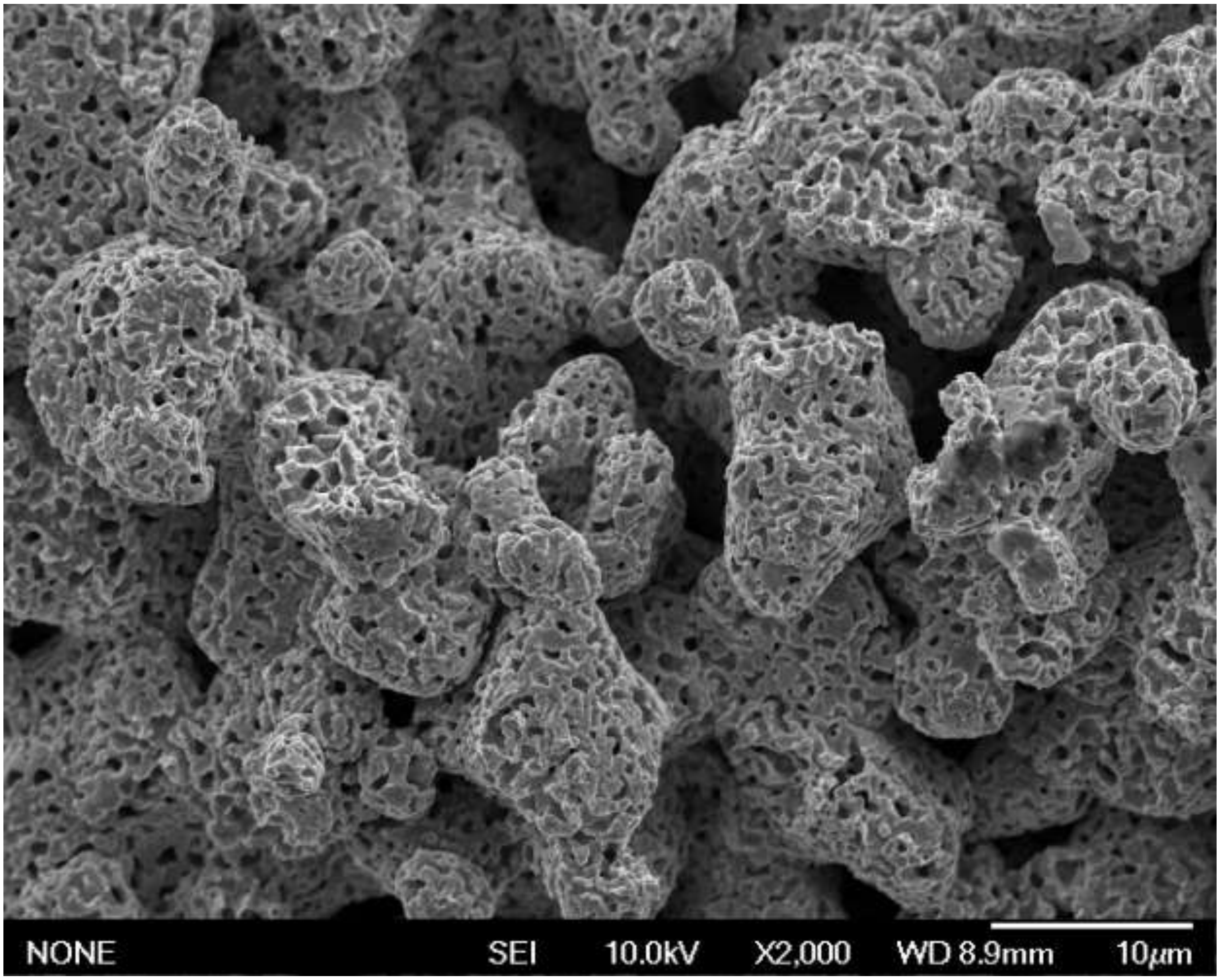

Supplementary Figure S12| Additional and larger viewing field SEM images of porous NiO pellets after NH_3 reduction.

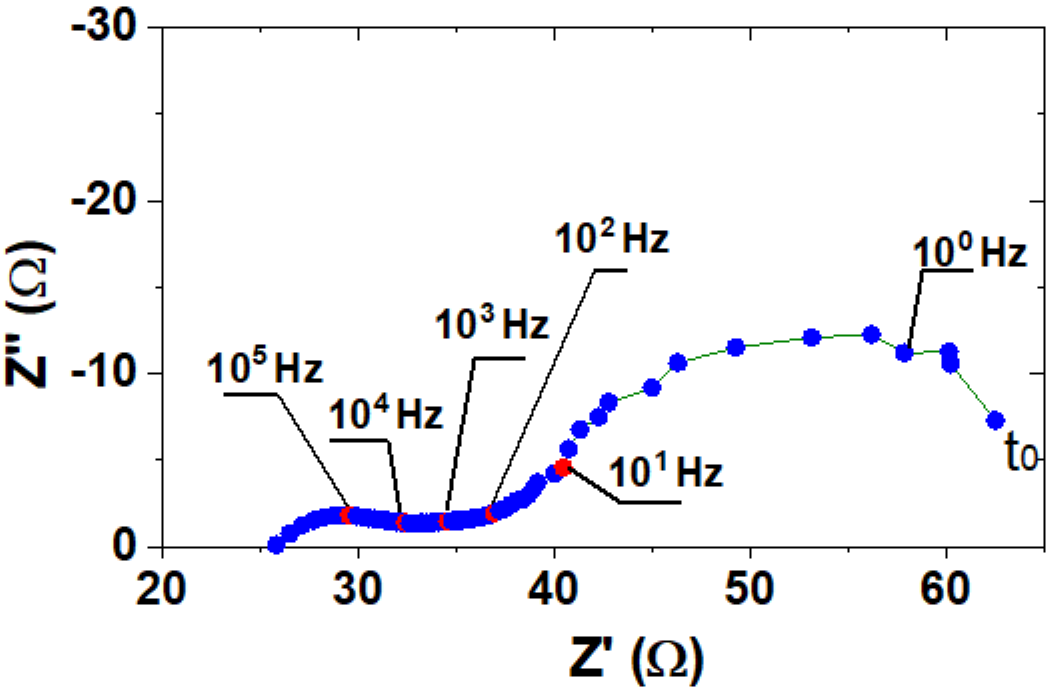

Supplementary Figure S13| Full electrochemical impedance spectroscopy (EIS) of a porous BCZYYb pellet (with 1 wt.% NiO) under N_2 flow and before switching to H_2 and NH_3.

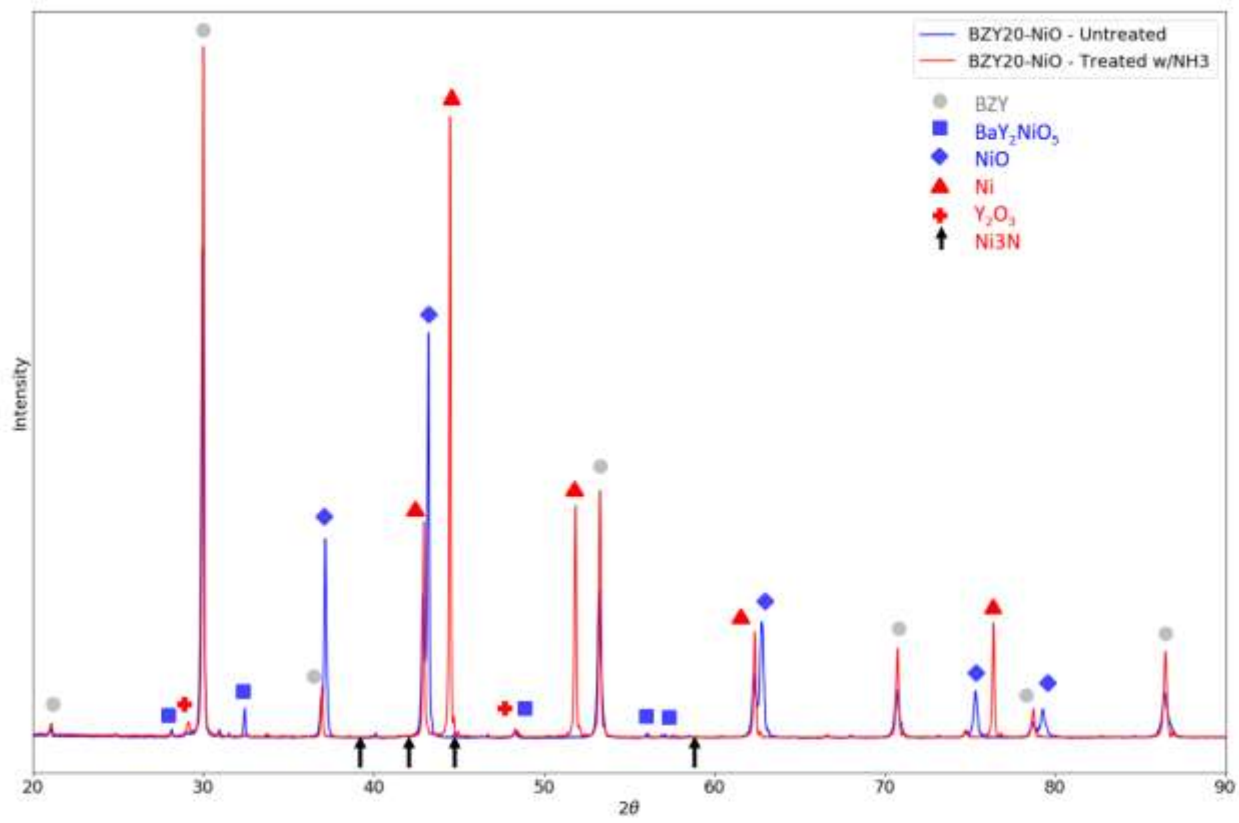

Supplementary Figure S14| Anode nitridation verification experiment with exposure to NH_3 gas. Little evidence for formation of Ni_3N under the testing conditions.

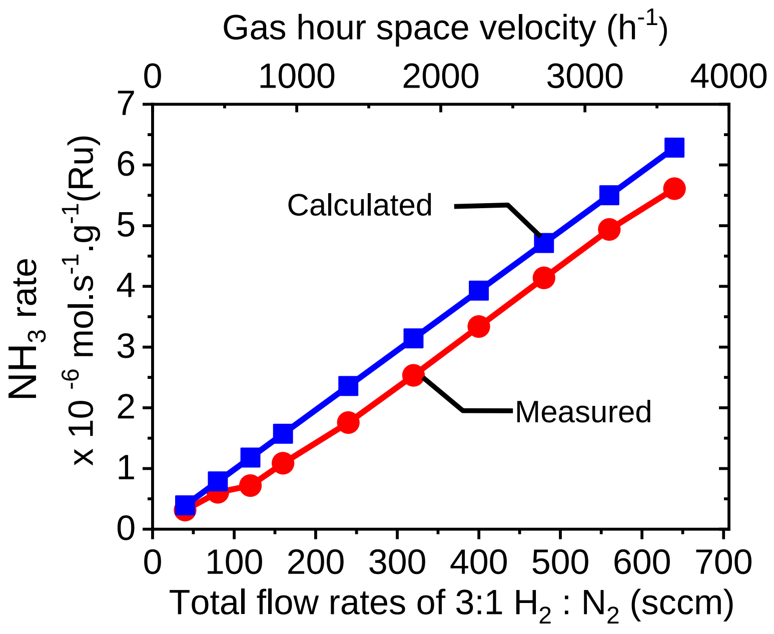

Supplementary Figure S15| Measured ammonia production rates as a function of total flow rate of 3:1 H_2 to N_2 gas mixture with comparison to equilibrium calculation. Reactor temperature at 400 ^{o}C.

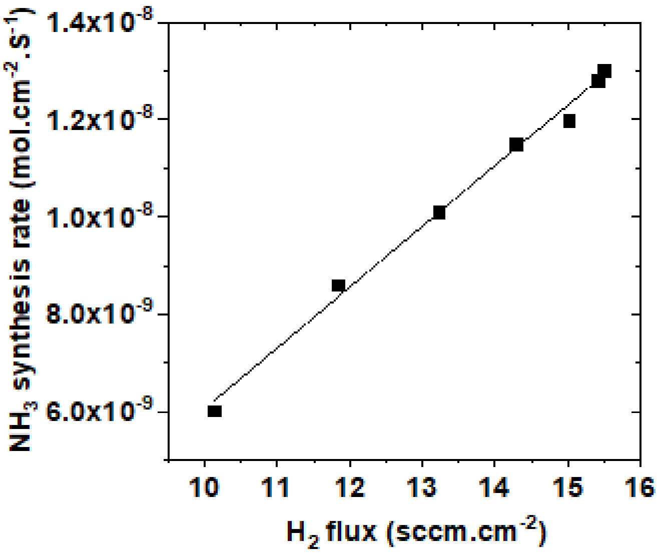

Supplementary Figure S16|Effect of hydrogen flux through the protonic-ceramic electrolyzer on ammonia-synthesis rate.

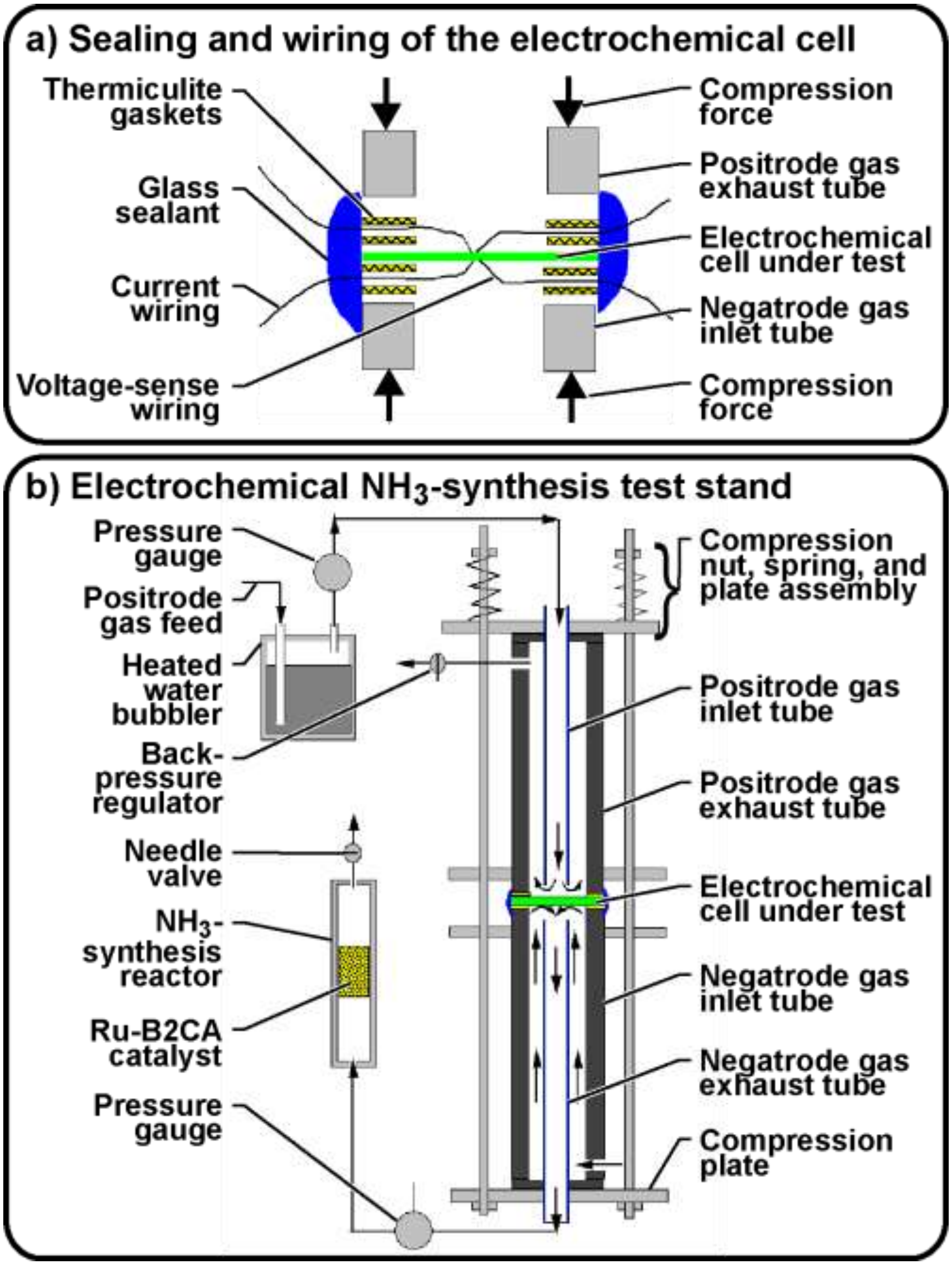

Supplementary Figure S17| Illustrations of pressure test. **a**, packaging and sealing of the high pressure protonic-ceramic membrane-electrode assembly within **b**, electrochemical test stand and downstream ammonia-synthesis reactor.

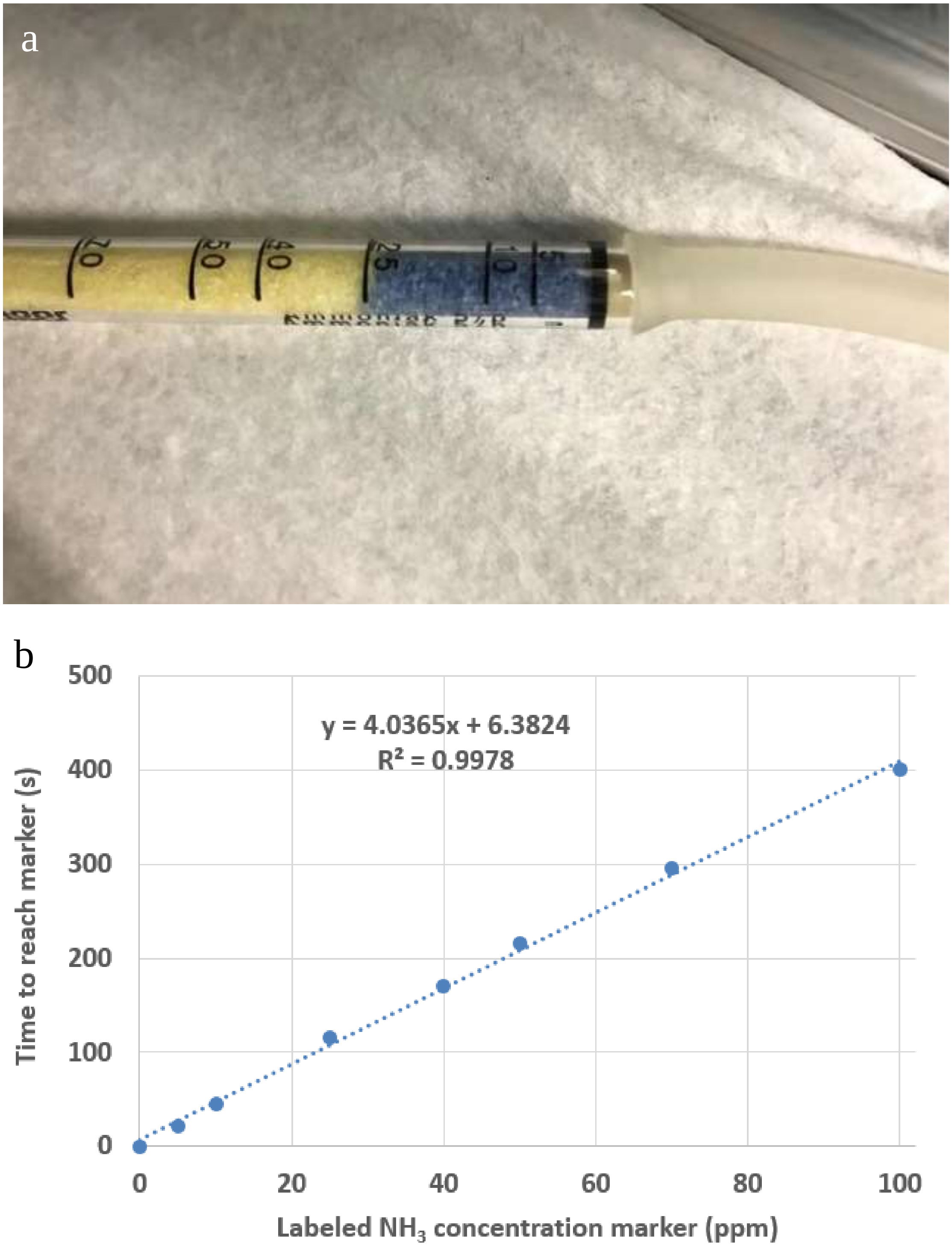

Supplementary Figure S18| Ammonia detection and calibration. **a**, a tested Drager tube showing the distinct color of NH_3 absorbed region (blue) and non-absorbed region (yellow) **b**, linear plot of time reaching each specific marker position versus labelled marker to using 10 ppm NH_3 in N_2 standard gas.

Supplementary references

1 Yang, J. *et al.* A stability study of Ni/Yttria-stabilized zirconia anode for direct ammonia solid oxide fuel cells. *ACS Applied Materials & Interfaces* **7**, 28701-28707, (2015).

2 Guangyao, M., Cairong, J., Jianjun, M., Qianli, M. & Xingqin, L. Comparative study on the performance of a SDC-based SOFC fueled by ammonia and hydrogen. *Journal of Power Sources* **173**, 189-193, (2007).

3 Aoki, Y. *et al.* High-Efficiency Direct Ammonia Fuel Cells Based on BaZr0.1Ce0.7Y0.2O3−δ/Pd Oxide-Metal Junctions. **2**, 1700088, (2018).

4 Zhang, L., Cong, Y., Yang, W. & Lin, L. A Direct Ammonia Tubular Solid Oxide Fuel Cell. *Chinese Journal of Catalysis* **28**, 749-751, (2007).

5 Maffei, N., Pelletier, L. & McFarlan, A. A high performance direct ammonia fuel cell using a mixed ionic and electronic conducting anode. *Journal of Power Sources* **175**, 221-225, (2008).

6 Ma, Q. *et al.* A high-performance ammonia-fueled SOFC based on a YSZ thin-film electrolyte. *Journal of Power Sources* **164**, 86-89, (2007).

7 Zhang, L. & Yang, W. Direct ammonia solid oxide fuel cell based on thin proton-conducting electrolyte. *Journal of Power Sources* **179**, 92-95, (2008).

8 Liu, M. *et al.* Direct liquid methanol-fueled solid oxide fuel cell. *Journal of Power Sources* **185**, 188-192, (2008).

9 Liu, L. *et al.* Improved performance of ammonia-fueled solid oxide fuel cell with SSZ thin film electrolyte and Ni-SSZ anode functional layer. *International Journal of Hydrogen Energy* **37**, 10857-10865, (2012).

10 Lin, Y. *et al.* Proton-conducting fuel cells operating on hydrogen, ammonia and hydrazine at intermediate temperatures. *International Journal of Hydrogen Energy* **35**, 2637-2642, (2010).

11 Ma, Q., Peng, R., Tian, L. & Meng, G. Direct utilization of ammonia in intermediate-temperature solid oxide fuel cells. *Electrochemistry Communications* **8**, 1791-1795, (2006).

12 Hawtof, R. *et al.* Catalyst-free, highly selective synthesis of ammonia from nitrogen and water by a plasma electrolytic system. *Science Advances* **5**, eaat5778, (2019).

13 Li, Z., Liu, R., Xie, Y., Feng, S. & Wang, J. A novel method for preparation of doped Ba3(Ca1.18Nb1.82)O9−δ: Application to ammonia synthesis at atmospheric pressure. *Solid State Ionics* **176**, 1063-1066, (2005).

14 Murakami, T., Nishikiori, T., Nohira, T. & Ito, Y. Investigation of Anodic Reaction of Electrolytic Ammonia Synthesis in Molten Salts Under Atmospheric Pressure. **152**, D75-D78, (2005).

15 Chen, C. & Ma, G. Preparation, proton conduction, and application in ammonia synthesis at atmospheric pressure of La0.9Ba0.1Ga1–xMgxO3–α. *Journal of Materials Science* **43**, 5109-5114, (2008).

16 Murakami, T., Nohira, T., Goto, T., Ogata, Y. H. & Ito, Y. Electrolytic ammonia synthesis from water and nitrogen gas in molten salt under atmospheric pressure. *Electrochimica Acta* **50**, 5423-5426, (2005).

17 Xie, Y.-H. *et al.* Preparation of La1.9Ca0.1Zr2O6.95 with pyrochlore structure and its application in synthesis of ammonia at atmospheric pressure. *Solid State Ionics* **168**, 117-121, (2004).

18 Xu, G., Liu, R. & Wang, J. Electrochemical synthesis of ammonia using a cell with a Nafion membrane and SmFe0.7Cu0.3−xNixO3 (x = 0−0.3) cathode at atmospheric pressure and lower temperature. *Science in China Series B: Chemistry* **52**, 1171-1175, (2009).

19 Vasileiou, E. *et al.* Electrochemical enhancement of ammonia synthesis in a BaZr0.7Ce0.2Y0.1O2.9 solid electrolyte cell. *Solid State Ionics* **288**, 357-362, (2016).

20 Liu, R. & Xu, G. Comparison of Electrochemical Synthesis of Ammonia by Using Sulfonated Polysulfone and Nafion Membrane with Sm1.5Sr0.5NiO4. *Chinese Journal of Chemistry* **28**, 139-142, (2010).

21 Kyriakou, V., Garagounis, I., Vourros, A., Vasileiou, E. & Stoukides, M. An Electrochemical Haber-Bosch Process. *Joule* **4**, 142-158, (2020).

22 Xu, G. & Liu, R. Sm1.5Sr0.5MO4 (M=Ni, Co, Fe) Cathode Catalysts for Ammonia Synthesis at Atmospheric Pressure and Low Temperature. *Chinese Journal of Chemistry* **27**, 677-680, (2009).

23 Zhang, Z., Zhong, Z. & Liu, R. Cathode catalysis performance of SmBaCuMO5+δ (M=Fe, Co, Ni) in ammonia synthesis. *Journal of Rare Earths* **28**, 556-559, (2010).

24 Liu, R.-Q., Xie, Y.-H., Wang, J.-D., Li, Z.-J. & Wang, B.-H. Synthesis of ammonia at atmospheric pressure with Ce0.8M0.2O2−δ (M=La, Y, Gd, Sm) and their proton conduction at intermediate temperature. *Solid State Ionics* **177**, 73-76, (2006).

25 Lan, R., Irvine, J. T. S. & Tao, S. Synthesis of ammonia directly from air and water at ambient temperature and pressure. *Scientific Reports* **3**, 1145, (2013).

26 Lan, R. & Tao, S. Electrochemical synthesis of ammonia directly from air and water using a Li+/H+/NH4+ mixed conducting electrolyte. *RSC Advances* **3**, 18016-18021, (2013).

27 Amar, I. A., Lan, R. & Tao, S. Synthesis of ammonia directly from wet nitrogen using a redox stable La0.75Sr0.25Cr0.5Fe0.5O3−δ–Ce0.8Gd0.18Ca0.02O2−δ composite cathode. *RSC Advances* **5**, 38977-38983, (2015).

28 Amar, I. A., Lan, R., Petit, C. T. G. & Tao, S. Electrochemical Synthesis of Ammonia Based on Co3Mo3N Catalyst and LiAlO2–(Li,Na,K)2CO3 Composite Electrolyte. *Electrocatalysis* **6**, 286-294, (2015).

29 Amar, I. A., Lan, R., Petit, C. T. G., Arrighi, V. & Tao, S. Electrochemical synthesis of ammonia based on a carbonate-oxide composite electrolyte. *Solid State Ionics* **182**, 133-138, (2011).

30 Amar, I., Lan, R., Petit, C. & Tao, S. *Electrochemical Synthesis of Ammonia Using Fe3Mo3N Catalyst and Carbonate-Oxide Composite Electrolyte*. Vol. 10 (2015).

31 Wang, B. H., Wang, J. D., Liu, R., Xie, Y. H. & Li, Z. J. Synthesis of ammonia from natural gas at atmospheric pressure with doped ceria–Ca3(PO4)2–K3PO4 composite electrolyte and its proton conductivity at intermediate temperature. *Journal of Solid State Electrochemistry* **11**, 27-31, (2007).

32 Li, Z. *et al.* Preparation of double-doped BaCeO3 and its application in the synthesis of ammonia at atmospheric pressure. *Science and Technology of Advanced Materials* **8**, 566-570, (2007).

33 Lan, R., Alkhazmi, K. A., Amar, I. A. & Tao, S. Synthesis of ammonia directly from wet air at intermediate temperature. *Applied Catalysis B: Environmental* **152-153**, 212-217, (2014).

34 Amar, I. A., Lan, R. & Tao, S. Electrochemical Synthesis of Ammonia Directly from Wet N2 Using La0.6Sr0.4Fe0.8Cu0.2O3-δ-Ce0.8Gd0.18Ca0.02O2-δ Composite Catalyst. **161**, H350-H354, (2014).

35 Lan, R., Alkhazmi, K. A., Amar, I. A. & Tao, S. Synthesis of ammonia directly from wet air using Sm0.6Ba0.4Fe0.8Cu0.2O3−δ as the catalyst. *Faraday Discussions* **182**, 353-363, (2015).

36 Amar, I. A., Lan, R., Humphreys, J. & Tao, S. Electrochemical synthesis of ammonia from wet nitrogen via a dual-chamber reactor using La0.6Sr0.4Co0.2Fe0.8O3−δ-Ce0.8Gd0.18Ca0.02O2−δ composite cathode. *Catalysis Today* **286**, 51-56, (2017).

37 Chen, C. & Ma, G. Proton conduction in BaCe1−xGdxO3−α at intermediate temperature and its application to synthesis of ammonia at atmospheric pressure. *Journal of Alloys and Compounds* **485**, 69-72, (2009).

38 Marnellos, G. & Stoukides, M. Ammonia Synthesis at Atmospheric Pressure. *Science* **282**, 98, (1998).

39 Lan, R., Alkhazmi, K. A., Amar, I. A. & Tao, S. Synthesis of ammonia directly from wet air using new perovskite oxide La0.8Cs0.2Fe0.8Ni0.2O3-δ as catalyst. *Electrochimica Acta* **123**, 582-587, (2014).

40 Wang, W. B. *et al.* Ammonia synthesis at atmospheric pressure using a reactor with thin solid electrolyte BaCe0.85Y0.15O3−α membrane. *Journal of Membrane Science* **360**, 397-403, (2010).

41 Yun, D. S. *et al.* Electrochemical ammonia synthesis from steam and nitrogen using proton conducting yttrium doped barium zirconate electrolyte with silver, platinum, and lanthanum strontium cobalt ferrite electrocatalyst. *Journal of Power Sources* **284**, 245-251, (2015).

42 Amar, I. A. *et al.* Electrochemical synthesis of ammonia from N2 and H2O based on (Li,Na,K)2CO3–Ce0.8Gd0.18Ca0.02O2−δ composite electrolyte and CoFe2O4 cathode. *International Journal of Hydrogen Energy* **39**, 4322-4330, (2014).

43 Wang, W. B. *et al.* Microstructures and proton conduction behaviors of Dy-doped BaCeO3 ceramics at intermediate temperature. *Solid State Ionics* **181**, 667-671, (2010).

44 Kumari, S., Pishgar, S., Schwarting, M. E., Paxton, W. F. & Spurgeon, J. M. Synergistic plasma-assisted electrochemical reduction of nitrogen to ammonia. *Chemical Communications* **54**, 13347-13350, (2018).

45 Li, Z.-J., Liu, R.-Q., Wang, J.-D., Xie, Y.-H. & Yue, F. Preparation of BaCe0.8Gd0.2O3−δ by the citrate method and its application in the synthesis of ammonia at atmospheric pressure. *Journal of Solid State Electrochemistry* **9**, 201-204, (2005).

46 Vasileiou, E., Kyriakou, V., Garagounis, I., Vourros, A. & Stoukides, M. Ammonia synthesis at atmospheric pressure in a BaCe0.2Zr0.7Y0.1O2.9 solid electrolyte cell. *Solid State Ionics* **275**, 110-116, (2015).

47 Liu, J. *et al.* Proton conduction at intermediate temperature and its application in ammonia synthesis at atmospheric pressure of BaCe1−xCaxO3−α. *Journal of Materials Science* **45**, 5860-5864, (2010).

48 Kim, J. N. *et al.* Electrochemical Synthesis of Ammonia from Water and Nitrogen using a Pt/GDC/Pt Cell. *Korean chemical engineering research* **52**, 58-62, (2014).

49 Wang, X., Yin, J., Xu, J., Wang, H. & Ma, G. Chemical Stability, Ionic Conductivity of BaCe0.9−xZrxSm0.10O3−α and Its Application to Ammonia Synthesis at Atmospheric Pressure. *Chinese Journal of Chemistry* **29**, 1114-1118, (2011).

50 Otomo, J., Noda, N. & Kosaka, F. Electrochemical Synthesis of Ammonia with Proton Conducting Solid Electrolyte Fuel Cells at Intermediate Temperatures. **68**, 2663-2670, (2015).
51 Zhang, M., Xu, J. & Ma, G. Proton conduction in BaxCe0.8Y0.2O3−α + 0.04ZnO at intermediate temperatures and its application in ammonia synthesis at atmospheric pressure. *Journal of Materials Science* **46**, 4690-4694, (2011).
52 Zhou, F. *et al.* Electro-synthesis of ammonia from nitrogen at ambient temperature and pressure in ionic liquids. *Energy & Environmental Science* **10**, 2516-2520, (2017).
53 Zhang, F. *et al.* Proton conduction in La0.9Sr0.1Ga0.8Mg0.2O3−α ceramic prepared via microemulsion method and its application in ammonia synthesis at atmospheric pressure. *Materials Letters* **61**, 4144-4148, (2007).
54 Kordali, V., Kyriacou, G. & Lambrou, C. Electrochemical synthesis of ammonia at atmospheric pressure and low temperature in a solid polymer electrolyte cell. *Chemical Communications*, 1673-1674, (2000).
55 Ouzounidou, M., Skodra, A., Kokkofitis, C. & Stoukides, M. Catalytic and electrocatalytic synthesis of NH3 in a H+ conducting cell by using an industrial Fe catalyst. *Solid State Ionics* **178**, 153-159, (2007).
56 Guo, Y. *et al.* Preparation via microemulsion method and proton conduction at intermediate-temperature of BaCe1−xYxO3−α. *Electrochemistry Communications* **11**, 153-156, (2009).
57 Wang, J.-D., Xie, Y.-H., Zhang, Z.-F., Liu, R.-Q. & Li, Z.-J. Protonic conduction in Ca2+-doped La2M2O7 (M=Ce, Zr) with its application to ammonia synthesis electrochemically. *Materials Research Bulletin* **40**, 1294-1302, (2005).
58 Skodra, A. & Stoukides, M. Electrocatalytic synthesis of ammonia from steam and nitrogen at atmospheric pressure. *Solid State Ionics* **180**, 1332-1336, (2009).
59 Yoo, C.-Y. *et al.* Role of protons in electrochemical ammonia synthesis using solid-state electrolytes. *ACS Sustainable Chemistry & Engineering* **5**, 7972-7978, (2017).
60 Neese, F. The Yandulov/Schrock cycle and the nitrogenase reaction: pathways of nitrogen fixation studied by density functional theory. *Angewandte Chemie International Edition* **45**, 196-199, (2006).
61 Singh, A. R. *et al.* Electrochemical ammonia synthesis—the selectivity challenge. *ACS Catalysis* **7**, 706-709, (2017).
62 Skúlason, E. *et al.* A theoretical evaluation of possible transition metal electro-catalysts for N2 reduction. *Physical Chemistry Chemical Physics* **14**, 1235-1245, (2012).
63 Foster, S. L. *et al.* Catalysts for nitrogen reduction to ammonia. *Nature Catalysis* **1**, 490-500, (2018).
64 Garden, A. L. & Skúlason, E. The Mechanism of Industrial Ammonia Synthesis Revisited: Calculations of the Role of the Associative Mechanism. *The Journal of Physical Chemistry C* **119**, 26554-26559, (2015).
65 Stucke, N., Flöser, B. M., Weyrich, T. & Tuczek, F. Nitrogen Fixation Catalyzed by Transition Metal Complexes: Recent Developments. *European Journal of Inorganic Chemistry* **2018**, 1337-1355, (2018).
66 Montoya, J. H., Tsai, C., Vojvodic, A. & Nørskov, J. K. The challenge of electrochemical ammonia synthesis: a new perspective on the role of nitrogen scaling relations. *ChemSusChem* **8**, 2180-2186, (2015).
